# Quantification of Urticating Setae of Oak Processionary Moth (*Thaumetopoea processionea*) and Exposure Hazards

**DOI:** 10.3390/ijerph22091361

**Published:** 2025-08-29

**Authors:** Paula Halbig, Horst Delb, Axel Schopf

**Affiliations:** 1 Department of Forest Protection, Forest Research Institute Baden-Wuerttemberg, Wonnhaldestr. 4, 79100 Freiburg, Germany; 2 Institute of Forest Entomology, Forest Pathology and Forest Protection, Department of Ecosystem Management, Climate and Biodiversity, BOKU University, Peter-Jordan-Str. 82/I, 1190 Vienna, Austria

**Keywords:** aeroallergen dispersion, biohazard, contamination, Lepidoptera, Notodontidae, *Quercus*, urticating caterpillar hairs

## Abstract

Potential climatic and land-use changes may favor an increase in the population densities and range expansion of oak processionary moth (OPM) in Central and Western Europe in the future. This could lead to more significant threats to human and animal health, caused by the urticating setae released by OPM larvae, and more severe oak defoliation by the larvae. To cope with the public health issue, a basis for OPM hazard assessment and management was created by quantifying the setae formation potential of OPM. While a single larva forms ca. 857,000 setae during its lifespan, a single infested oak tree may be contaminated with up to 10–24 billion (10^9^) setae during an OPM outbreak. Moreover, the possible setae contamination threat to humans through airborne setae dispersal was studied in worst-case exposure simulations in the field. The highest airborne setae concentration was straight downwind, but turbulences up to 150° from the air flow were observed. The findings of this study will improve biohazard quantification as a basis for decision-making on preventive or mechanical control measures and enable an effective protection of human health. This study provides applicable information to derive warnings and recommendations for the public, as well as land managers and authorities.

## 1. Introduction

Worldwide, various processionary species are socio-economically important due to their hazards to human and animal health, as well as their occurrence as plant pests. Some tend towards outbreaks on coniferous and broadleaved trees or shrubs [1]. Battisti et al. [2] presented a review of the species, their distribution in Europe, Africa, Asia and Australia, their host plants and the stage-specific formation of urticating setae, and assessed the potential effects of global change on the species’ occurrence.

In Central Europe, the population densities of oak processionary moth (OPM, *Thaumetopoea processionea* L., Lep., Notodontidae) have risen, and the spatial distribution of this native insect has extended since about 1990. Furthermore, OPM was accidentally introduced into the United Kingdom in 2006, where it is currently spreading in the southeast of England at a rate of ca. 1 km per year [3,4]. Consequently, its presence and importance as a feared biohazard and severe defoliator of oak trees, *Quercus* spp., have increased (e.g., [4,5,6,7,8,9,10]). Thousands of hectares of forest have been infested in Germany during OPM outbreaks (e.g., [11,12,13]). The regulative mechanisms of OPM population dynamics are not yet completely understood. Improved climatic suitability in Central and Western Europe in recent decades probably led to higher OPM population densities at its range limits and recolonizations of former habitats [8]. Moreover, natural forest transition, forest conversion and augmentations of urban green spaces with oak trees, which are considered to be more heat- and drought-tolerant, create additional habitats for OPM and thus aggravate OPM-related hazards (e.g., [14,15,16,17,18,19]). OPM-infested oaks pose a serious hazard of contamination of humans and animals with setae (e.g., [9,20,21,22,23]). Therefore, affected forests, copses and urban green spaces are routinely classified as hazardous to public health by local authorities in specific regulations and must be cordoned off or treated with biocides (e.g., [3,9,24,25,26,27,28]). Forestry operations are postponed or avoided to prevent workers from being exposed to setae (cf. [29]).

Setae are bristle- or hair-like structures on organisms, such as plants, fungi, diatoms (e.g., [30,31,32]) and animals, such as annelids, crustaceans, arachnids, insects, lizards (e.g., [2,33,34,35,36,37,38]). Depending on the taxon and habitat of the organisms, setae have different structures and functions. In insects, they are cuticular protuberances and serve for, e.g., mechanoreception, defense, camouflage, pheromone dispersal, locomotion and attachment (e.g., [35,39]). Battisti et al. [40] give an overview of urticating hairs in arthropods. Setae of OPM and other Lepidopterans, especially from the genus *Thaumetopoea* and further genera feeding gregariously on trees, are urticating [1,2,40,41,42,43]. They are generally considered to act as protection against predatory vertebrates such as birds and small mammals, which was first suggested in basic studies (e.g., [41,44,45,46,47]), and which was confirmed in more recent studies (e.g., [40,42,43,48]). The larvae of processionary species can release the setae into the air so that they form a protective cloud around the larval colony and thus serve as strategic rather than tactical defense [24,49]). However, setae do not provide 100% protection against vertebrate predators. Nevertheless, some bird species feed on larvae and pupae of OPM and pine processionary moth (PPM, *Thaumetopoea pityocampa*), e.g., *Cuculus canorus*, and *Upupa epops,* as well as Corvidae, Oriolidae, Paridae, Passeridae and Picidae [4,20,44,50,51,52]. Furthermore, mice were observed to feed on OPM pupation tents at the lower tree trunk [52].

In OPM, which is univoltine and starts its larval development in early spring [53,54,55,56,57], the larvae form setae from the third instar (L3) onwards. Throughout their entire development, the larvae live gregariously and join in characteristic processions as they move along the tree. With advancing larval age from L3 to the last instar, L6, the number of setae per OPM larva increases (own observations; [58]; see also [59]). While the total number of setae per OPM larva has not yet been determined, a single L5 larva of PPM can produce at least one million setae [60,61].

In addition to the OPM larvae themselves, the exuviae and the cocoons in the tents (“nests”) spun by the larvae for molting and pupation on branches or the trunk of the host trees carry and contain setae, respectively. (e.g., [4,46,53,54]; see also Supplementary Data 2.3.1: Figure S2). The tents are made of silk and comprise larval frass (own observations; e.g., [4]). The setae of processionary larvae are released during larval movement due to their easy detachment from the integument by air currents or by mechanical stimulation [20,39,46,58,60,61,62,63]. From cocoons and exuviae, the setae are released passively by wind gusts.

**Figure 1:**
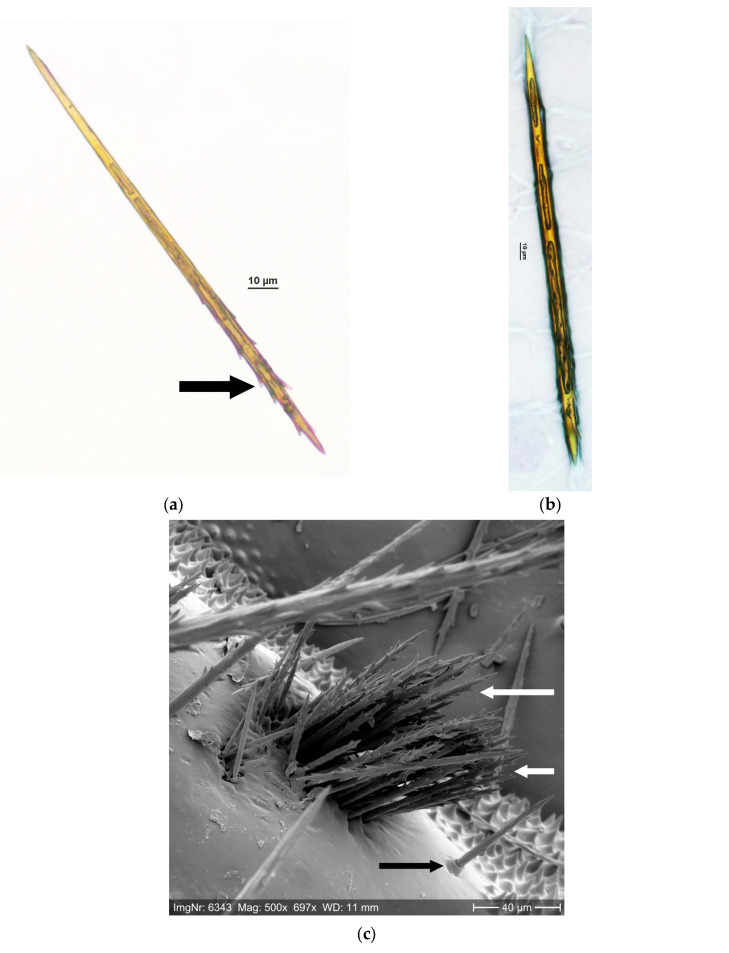

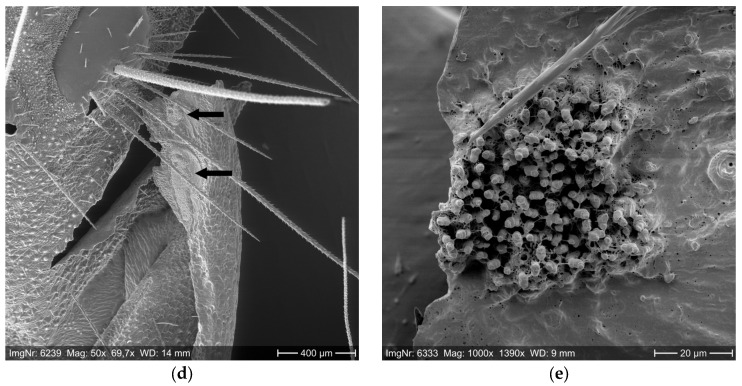
(**a**,**b**) OPM setae: chitin coat with cavity inside, barbs at the distal end (arrow); transmitted-light microscope photos (NIKON Eclipse E800 connected to NIKON Photo Head V-TP Multi-Point Sensor System; NIKON CORPORATION, Tokyo, Japan); (**c**) OPM setae in the mirror of an OPM L4 larva; barbs at the distal end (white arrows); true hair with collar at the base (black arrow); (**d**) lower side of two setae-carrying mirrors (arrows) of an OPM L5 exuvia; (**e**) trichogen cells on the lower side of a setae-carrying mirror of an OPM L4 exuvia.

In processionary species, true setae are tubular but closed at both ends, and they are neither fixedly attached to the integument nor neurally connected [40,59] (Figure 1a,b). The setae have barbs at their distal end (Figure 1a,c) (e.g., [47,49,60,64,65,66]) and range in length from approximately 80 to 300 µm and in diameter from 3 to 11 µm [49,67]. The setae are formed on the dorsal side of the abdominal segments in integument areas called “mirrors” due to their light reflectivity (e.g., [40,47,58,63,64]) (Figure 1c–e). The basis of the mirrors is trichogen cells embedded in the epidermis [40] (Figure 1e). The setae of processionary species consist of a chitin cover and urticating proteins inside (e.g., [40,42,47,68,69,70,71,72,73]) (Figure 1a,b). To understand the functional roles of the setae components, an examination of the variability in the venom profiles in different life stages and under different environmental conditions is necessary [69].

Humans and animals may be contaminated with OPM setae by touching larvae, exuviae and tents or the setae-contaminated surrounding vegetation or objects such as firewood, but most commonly through airborne dispersal of setae (e.g., [9,21,22,29,44,60], [73] and references therein, [74,75,76,77]). The setae proteins and allergens enter by intradermal injection [68].

Exposure to airborne setae and skin contact of humans and animals with *Thaumetopoea* setae leads to erucism, i.e., unspecific symptoms such as pruritus, contact dermatitis (caterpillar dermatitis), inflammations of the eyes, and respiratory signs (e.g., [9,21,22,44,60,74,78,79,80,81]). In the worst case, contact with setae can cause an anaphylactic shock [23,47,59,65,82]. The reactions are mechanically triggered by skin penetration and setae breaking, chemically by the release of urticating proteins and by chitin ([39,59,60,65,68,71], [46,69] and references therein). Repeated exposure to OPM and PPM can lead to sensitization with increasing reaction intensity, which indicates immunoglobulin E (IgE)-dependent allergies [23,72,81,83]. In addition to the allergic IgE antibody-mediated reactions, which can also be induced by various setae allergens, possibly toxic reactions or reactions other than IgE-mediated are effective [40,68,73] (see also [23]). Nonallergic and allergic mechanisms can overlap [23]. In regions where different processionary species are present, monosensitization of humans to only one species, but more frequently, polysensitization to the larval allergens of several species can occur [84].

In humans and animals, skin reactions usually occur immediately or up to 24 h after setae contact and may last for ca. 10–14 days (own observations; [21,47,60,79,81], [40] and references therein). The time lag between exposure and manifestation complicates relating the symptoms to the causal agent (see also [43]). The severity of human reactions depends on individual sensitivity and the routes of exposure [9,20,23,40,47,74]. Skin reactions often occur on face, hands, arms and neck, due to their predominant exposure ([21,60], cf. [29]). Currently, there is no specific medication for treating OPM envenomation [81].

The setae may keep their urticating potential for at least 12 years, accumulate in OPM-colonized areas, and thus constitute a persistent threat to human and animal health (e.g., [20,21,22,23,26,27,40,44,59,74,75]).

Setae can be spread via air currents up to several hundreds of meters (e.g., [21,24,59,60,78]). The concentration of setae released into the air and their spatio-temporal airborne dispersion can be measured by means of traps. Passive traps are adhesive-coated surfaces on which airborne setae settle [85,86]. Due to the similar magnitude of sedimentation velocity and source location, aerial dispersal of setae is comparable to pollen [67]. Hence, pollen or spore samplers with active air suction, such as Hirst–Burkard traps [87,88], operate successfully in sampling airborne setae [29,41,78].

Information on setae characteristics and aerial setae spread, linked to the processionaries’ population density and phenology, can be used in modeling and spatio-temporal health hazard assessment. This was implemented in “URTIRISK”, an online tool for PPM in France [89]. Furthermore, the potential spatial dispersal capacity of OPM setae via air flow was modeled based on the setae morphology [24,67]. To estimate the phenological development of OPM, especially the occurrence of setae-carrying larvae, and to provide information on hazards and OPM management, the online early warning system “PHENTHAUproc” (Phenology modeling of *Thaumetopoea processionea*) was developed by Halbig et al. [57] and implemented in Germany by the German Meteorological Service [90].

OPM-related hazards are assumed to increase in future [4,91]. In view of climate change and the unpredictability of OPM infestations (e.g., [8,10,55,92]), appropriate strategies for OPM management are required, including the raising of public and professional awareness (e.g., [10,93,94]). This requirement is reinforced by the complex and sometimes indistinguishable regulatory responsibilities in public health and plant protection (e.g., [10,22,95]).

As a basis for health hazard assessment, the present study focused on OPM setae formation and contamination of humans. First, the total number of setae per larva was determined and extrapolated to the OPM colony and entire infested tree. Moreover, exposure simulation experiments were performed to investigate airborne setae dispersion in the field at close range to the setae source at ground level.

## 2. Materials and Methods

### 2.1. Setae Quantification

The total number of setae produced per OPM larva was determined to assess the airborne setae contamination potential. Five exuviae or larvae of each setae-carrying OPM instar from L3 to L6 were examined. In advance, the instar was determined by checking the number of setae-carrying mirrors and their distribution on the abdominal segments with a binocular microscope (NIKON SMZ 1500; NIKON CORPORATION, Tokyo, Japan). This method was considered more reliable than measuring the head capsule width, as the latter varies with larval size depending on the environmental conditions such as the nutritional status (own observations; [96]). The samples originated from *Q. robur* from the field from Lorraine, France, and from the Upper Rhine Valley, Germany (Freiburg-St. Georgen and Schallstadt/Mengen), as well as from a semi-field (Forest Research Institute Baden-Wuerttemberg (FVA), Freiburg; for geographic coordinates, see Supplementary Data 1, Table S1; for details on sample collection, see Supplementary Data 2.1). The leaf quality of the host trees from which the samples were collected was not analyzed. The within-instar variability in the number of setae per larva was indicated.

For each sample (larva or exuvia), the entire setae-carrying area of the mirrors was measured, and the number of setae per mm^2^ mirror area was calculated. To prepare for scanning electron microscopy (SEM), the setae were mechanically removed from the mirrors in 70% ethanol to bare the entire mirror area so that the setae sockets (pits) were visible. Then, the dorsal skin with the mirrors was cut out and dried in 99% ethanol first and subsequently in hexamethyldisilazane (HMDS). After drying, the samples were attached to specimen stubs and sputter-coated with gold-palladium for 2 min (EDWARDS LTD., Burgess Hill, UK).

The SEM photos were taken with 50–1000× magnification on a ZEISS DSM 940A (CARL ZEISS AG, Oberkochen, Germany) equipped with a DISS5 unit (POINT ELECTRONIC GMBH, Halle, Germany) at the University of Freiburg, Chair of Forest Entomology and Forest Protection. The basic suitability of SEM photos for the determination of the number of OPM setae was proven in previous studies at the University of Freiburg. This method has been established by Ducombs et al. [60] and Lamy et al. [61] for PPM and for *Ochrogaster lunifer* by Perkins et al. [76].

The SEM photos were used for measurement of the setae-carrying area in dorsal and dorso-lateral mirrors by means of NIS-Elements Imaging Software (NIKON CORPORATION, Tokyo, Japan). If sections of wide-spaced setae sockets were detected, i.e., with a lower number of setae per area, the area of these sections was measured separately. The total setae-carrying area of the mirrors, distinguished between close-set and wide-spaced sockets, was computed for each exuvia or larva (for specifics, see Supplementary Data 2.2.1).

The number of setae was determined in a standardized procedure by counting the setae sockets in the mirrors on the SEM photos. Four counting squares of 25 µm × 25 µm each were analyzed per mirror (for details, see Supplementary Data 2.2.1, Figure S1). For each sample (larva or exuvia), the mean setae number per 25 µm × 25 µm square (625 µm^2^) was calculated and then converted into the number of setae per mm^2^. Sections of close-set and wide-spaced setae sockets within the mirrors were distinguished. Finally, by multiplying the mean number of setae per mm^2^ and the total setae-carrying area (mm^2^) of the mirrors per sample, the total number of setae per sample was computed. Due to the sample size of 5 samples per instar, statistical analysis was omitted.

The mean number of setae per larva of the different instars L3–L6 was calculated to quantify the total number of setae produced by an OPM individual during its entire lifespan. Accordingly, the setae number per OPM colony was computed. For this extrapolation, only the data from field samples from the Freiburg-St. Georgen and Schallstadt/Mengen in the Upper Rhine Valley, Germany, were used, apart from L3, for which only semi-field samples were available (for geographic coordinates, see Supplementary Data 1, Table S1).

To estimate the number of setae per OPM colony, the number of individuals/cocoons per pupation tent was determined based on field samples. A part of the data was obtained from the work of Mühlfeit et al. [52] from nine different locations in Germany (for details, see Supplementary Data 2.3.2). Furthermore, pupation tents of various sizes, collected in Pötzleinsdorf, Vienna, Austria, in summer 2014, were dissected, and the volume “V” of each tent was calculated based on measuring of the three diameters “A, B, C”, according to a tri-axial ellipsoid:

(1) $$V=\frac{\pi }{6}\times A\times B\times C$$

To estimate the setae contamination potential emanating from an entire OPM-infested tree, a tent volume of 28 L per tree was used as an example for the extrapolation. This was the average tent volume per tree at Michaelisbruch in Brandenburg, Germany, in 2017 at the point of culmination of the local OPM outbreak (for geographic coordinates, see Supplementary Data 1, Table S1). The standard error (SE) of the mean and the 95% confidence interval were computed to show the potential range of a whole-tree setae contamination during an outbreak culmination. Details on the tent size classification and the estimation of the whole-tree tent volume are given in Supplementary Data 2.3.3 and Supplementary Data 2.3.4.

Furthermore, to show that setae synthesis in OPM larvae is food-independent and to establish a new laboratory-rearing method, OPM was reared on a diet which did not contain oak leaves or oak-derived substances (see Supplementary Data 3). The larval development was recorded, and the formation of setae was analyzed and compared to OPM from the field.

### 2.2. Airborne Setae Dispersion

Two exposure simulation experiments were performed to investigate airborne setae dispersion in the field on warm, dry and as-windless-as-possible summer days in 2014 and 2015. The weather conditions during these experiments are characterized in Section 3.2. The experiments were prepared by a preliminary study, which is described in Supplementary Data 4.3.

The study site was a forest opening of ca. 8 ha at Klingenbach, Austria, in an oak (*Q. petraea*, *Q. cerris*) and hornbeam (*Carpinus betulus*) forest (for geographic coordinates, see Supplementary Data 1, Table S1; for an aerial photo, see Supplementary Data 4.1.3, Figure S7). In the forest stands surrounding the opening in the southwest and east, the OPM population was in retrogradation with less than one pupation tent per tree and foliage loss due to OPM feeding below 5% per tree.

The setae source for these experiments was created from OPM larvae as well as molting and pupation tents, respectively, collected at different sites (details see Section 2.2.1 and Section 2.2.2). It was placed in front of a motorized blower (CRAMER LS 5000 SW, GREENWORKS TOOLS EUROPE GMBH, Weiterstadt, Germany, with HONDA GX 160, HONDA MOTOR Co., Ltd., Tokyo, Japan; Supplementary Data 4.1.3, Figure S8). Passive samplers, an active volumetric sampler (AMET, Velké Bílovice, Czech Republic) and a dummy, respectively, were exposed at close range to the setae source, i.e., up to 20 m distance and up to 2 m above ground level (AGL). Photos and details on the sampling equipment, as well as the methods of sampler screening, are shown in Supplementary Data 4.1, Figures S6A–D and S8A–D and Supplementary Data 4.2.1. Details on the assessment of the setae source magnitude and of the setae quantity, which was not released into the air during the experiments but remained on the exuviae, are given in Supplementary Data 4.2.2.

#### 2.2.1. Close Range, Circular Dispersion

**Figure 2:**
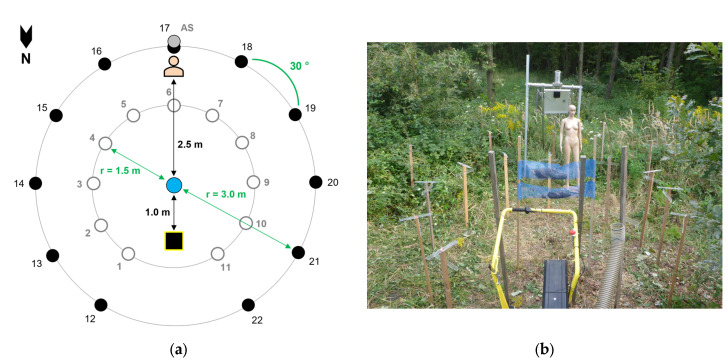
Experimental setup of the airborne setae exposure simulation with circular sampler arrangement and dummy. (**a**) Diagram (not true to scale): black square—blower, blue dot—setae source, white dots—large passive samplers in the inner circle (ID 1–11), black dots—large passive samplers in the outer circle (ID 12–22), grey dot—position of the active sampler (AS) and the small passive samplers, r—radius. (**b**) Photo (front to back): blower (black tube and yellow bail), setae source consisting of OPM tents in blue mesh bags, 2 circles of 11 passive samplers each on roof battens (samplers in the inner circle of samplers completely visible, outer circle partly displayed), dummy, active sampler on aluminum trestle and passive sampler; photo: Josef Pennerstorfer.

Conditions of a potential worst-case exposure scenario were simulated on 20 August 2014 from 16:00 to 18:00 Central European Summer Time (CEST). A total of twenty-two large passive samplers, two small passive samplers, one active sampler and a dummy were used (Figure 2). The large passive samplers consisted of a horizontal and a vertical plate of acrylic glass with a size of 40 mm × 300 mm each, which were coated with petroleum jelly and mounted on a roof batten at about 1.3 m AGL. They were arranged in two circles of ca. 300° each, at 1.5 m and 3.0 m distance, around the setae source in the circle center. Each circle consisted of 11 samplers (ID 1–11 and ID 12–22) placed in steps of ca. 30° to each other. The outer circle further comprised an active volumetric sampler at 2 m AGL, as well as a vertical and a horizontal small passive sampler of 76 mm × 26 mm each at 1.5 m AGL at position ID 17, straight in the blowing direction (Figure 2).

By means of a dummy equipped with 26 tapes of 30 mm × 70 mm each, the setae contamination of a person standing at 2.5 m distance downwind from the setae source was simulated. The foreside of each tape was coated with petroleum jelly. Twenty-four tapes were placed on the windward, front side of the dummy on different parts of the body. Two tapes were put on the back side of the upper body, on the nape of the neck and the back. Most tapes were placed on the head region and the arms, as these are often bare and thus more exposed to setae contamination.

The blower was positioned at 1 m distance in front of the setae source which consisted of 10 OPM tents. These were collected at Pötzleinsdorf in Vienna, Austria, in summer 2014 and frozen until the performance of this experiment (for geographic coordinates, see Supplementary Data 1, Table S1; for the tent volume and number of individuals per tent, see Supplementary Data 2.3.2, Table S3). The tents were put into mesh bags at about 1.5 m AGL with a mesh size of 5 mm × 5 mm. Wind speed was measured at different positions of the experimental setup at the start of the experiment, as well as 20 min and 40 min after the start, with a portable vane anemometer (EXAKT-ASDi, HÖNTZSCH GMBH & CO., KG, Waiblingen, Germany; Table 1).

The total setae amount which potentially contaminated the windward side of the dummy during the experiment was computed by extrapolation from the total body surface area and the setae catch of all tapes from the body front side, including the flanks. The body surface area of the dummy was calculated by means of the formula from Mosteller [97]. The dummy size corresponded approximately to a human of 180 cm height and 60 kg weight. The body surface area “a” is expressed as follows:

(2) $$a=\sqrt{\frac{l\times m}{3600}},$$

where “l” is the body height in cm, and “m” is the body mass in kg. The windward side of the dummy was assumed as half of the total body surface area. The setae contamination on the back side of the dummy was estimated based on the setae amount caught by the tapes on back and nape, and it was compared to the front side, i.e., belly and neck.

#### 2.2.2. Horizontal Setae Dispersion

**Figure 3:**
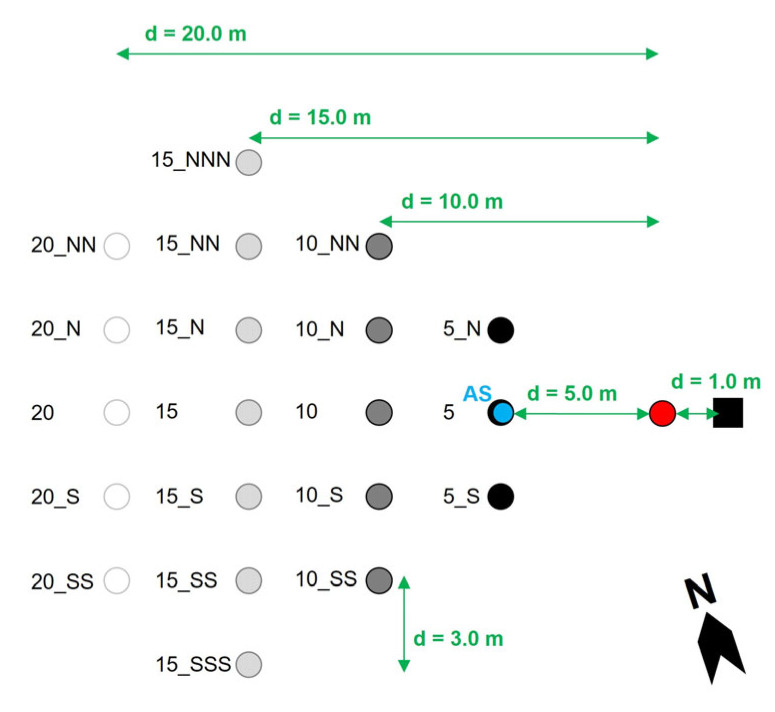
Experimental setup of the airborne setae exposure simulation with sampler arrangement in rows. d—distance: between the samplers and the setae source, between the individual samplers, between the blower and the setae source; black square—blower, red dot—setae source, blue dot—active sampler (AS); white, light grey, dark grey, black dots—passive samplers; diagram is not true to scale; for a photo of the experimental setup, see Supplementary Data 4.1.3, Figure S8C.

Another exposure simulation experiment was conducted on 22 June 2015 from 14:55 to 15:55 CEST. One active sampler and twenty passive samplers were installed in four rows with 5 m distance to each other (Figure 3; Supplementary Data 4.1.3, Figure S8C). The within-row distance between the samplers was 3 m. The samplers at the edges of the rows, e.g., positions 15_NNN and 20_NN, were placed at ca. 31° and 17° deviation, respectively, from the direction of the blower airstream. All passive samplers were mounted on roof battens at ca. 1.3 m AGL. Each sampler consisted of one small vertical slide of 25 mm × 80 mm in a pivoted beaker (for details, see Supplementary Data 4.1.2: Figure S6A,B) and one static large vertical slide of 40 mm × 300 mm. Horizontal slides were not used.

The setae source was placed at 5 m distance to the active sampler and at 1 m distance downwind from the blower (Figure 3, Supplementary Data 4.1.3: Figure S8C). It consisted of approximately 40–50 L of living OPM L6 larvae, single and in molting tents, as well as exuviae. They were collected near Fénétrange, Lorraine, France, on 19 June 2015, and transported cooled to the study site Klingenbach, Austria (for geographic coordinates, see Supplementary Data 1, Table S1). For the experiment, the OPM larvae and exuviae were put into mesh bags at 1.5–2.0 m AGL with a mesh size of 2 mm × 2 mm.

The gradient of setae catches with increasing row distance from the setae source was tested for significant differences using the non-parametric Kruskal–Wallis rank sum test, followed by the post hoc Dunn test with a 95% confidence interval [98,99,100]. *p*-values were adjusted by the Benjamini–Hochberg method. In addition, possible differences in the setae catches between the rows of samplers in diagonal direction were checked by the Kruskal–Wallis rank sum test, corresponding to the prevailing natural wind direction from south and south-west. This referred to the following diagonal rows: from sampler position 15_NNN to 5_N, from 15_NN to 5, continuing in the same way to the diagonal row from 20_SS to 15_SSS (Figure 3).

Before the experiment started, the wind speed at different positions at the study site was measured with a portable vane anemometer (EXAKT-ASDi, HÖNTZSCH GMBH & CO. KG, Germany), which revealed 12.6 m/s at the blower orifice, 6.5–7.5 m/s before the mesh bag and 2.0–3.5 m/s behind the mesh bag. The data of the natural wind speed were obtained from the official weather station Mattersburg, Austria, at 10 km distance (for geographic coordinates, see Supplementary Data 1, Table S2). Further meteorological data were measured by the weather station on site (for details, see Supplementary Data 4.1.4).

## 3. Results

### 3.1. Setae Quantification

**Figure 4:**
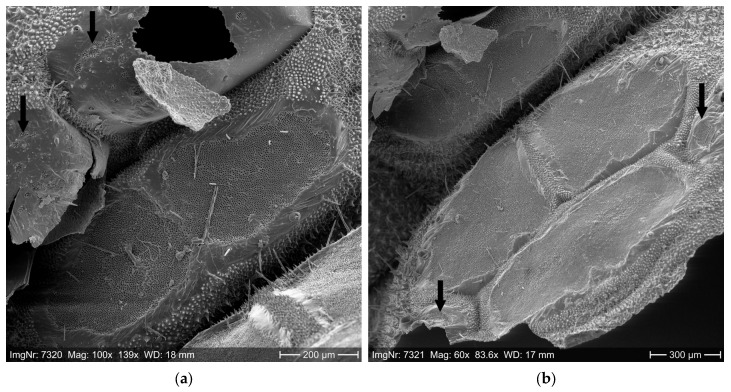

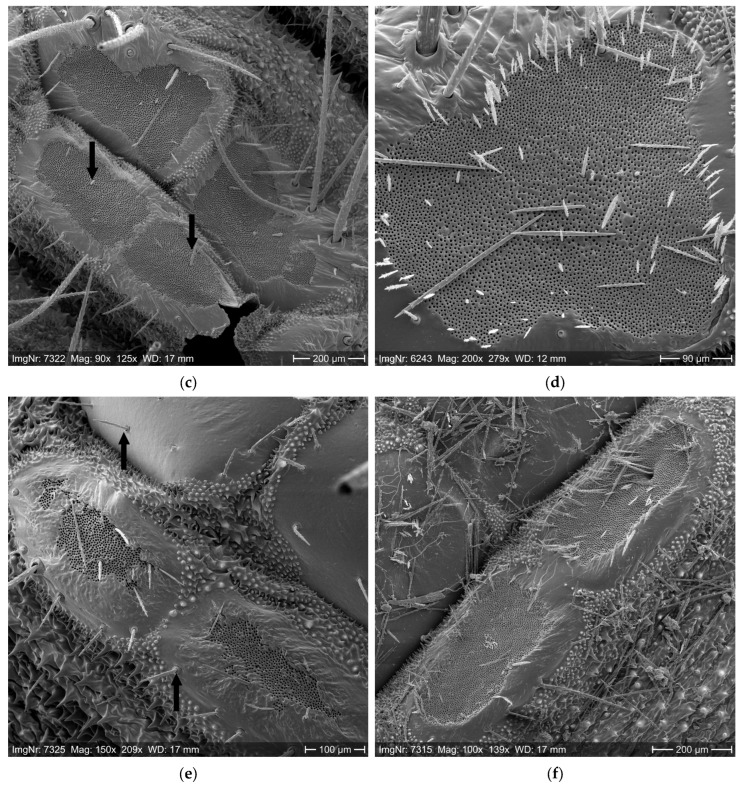
SEM photos of OPM mirrors from which the setae were mechanically detached. (**a**)—setae sockets (arrows) on the fore-mirrors of segment 10 (which is usually free of setae in the L5 instar) of an exceptionally large L5 larva from the field; (**b**)—dorso-lateral mirrors (arrows) of segment 11 of an exceptionally large L5 larva from the field; (**c**)—true hairs (arrows) within the mirror of segment 11 of an L5 larva; (**d**)—left fore-mirror with wide-spaced (mirror center) and close-set setae sockets (mirror edges) of segment 11 of an L5 larva; (**e**)—hind-mirrors of an L5 larva from the semi-field (small total size and small setae-carrying area with irregular edges, compared to Figure 4f), true hairs with collar at their base (arrows); (**f**)—hind-mirrors of segment 6 of an L5 larva from the field (large total size compared to Figure 4e; for geographic coordinates, see Supplementary Data 1, Table S1.

The number and size of the setae-carrying mirrors and, thus, the total number of setae per larva rise with increasing larval age from L3 to L6 (Table 2 and Table 3). Specifics on the distribution of the setae-carrying mirror area across the larval body and the setae arrangement within the mirrors are shown in Figure 4 and described in Supplementary Data 2.2.2. The number of setae per mm^2^ tended to increase with the larval age from L4 onwards. This applied to the mirror areas with close-set as well as wide-spaced sockets (Table 2). The size of the setae-carrying mirror area and, consequently, the total setae amount per larva depended on the sample origin from the field vs. semi-field and the concomitant larval body size (Figure 4e,f). The larvae reared under semi-field conditions were smaller and carried less setae than the larvae from the field. This discrepancy increased with larval age. While the OPM origin (field vs. semi-field) influenced the total mirror area per larva, it had no effect on the number of setae per mirror area (setae density).

The counting of OPM individuals per pupation tent and measuring the tent volume showed a linear relationship between these variables (see Supplementary Data 2.3.2, Figure S3). Besides the L6 exuviae, OPM pupation tents often contain exuviae shed by the preceding instars, especially L5 (own observations; [58]). The total setae amount per OPM colony comprises the setae formed by the L3–L6 larvae within one season (Table 4).

Based on the setae amount per mm^2^, the setae-carrying mirror area per larva, the number of OPM individuals per colony (pupation tent) and the mean tent volume per oak tree during an OPM outbreak, the extrapolation of the setae contamination potential in the field revealed:

- Each OPM larva produces ca. 857,000 setae during its entire lifespan (sum of the means for the instars L3–L6; ca. 834,900 (minimum); 882,900 (maximum); Table 2, Table 3).
- OPM pupation tents can reach volumes of ca. 0.2–12.0 L each, depending on OPM population density (see Supplementary Data 2.3.3).
- An OPM pupation tent with a volume of 1 L contains from 400 individuals according to Mühlfeit et al. [52] to 998 ± 246 individuals (mean ± SD) according to the counting in the present study. The first values refer to loose, less densely woven tents, whereas the latter values pertain to compact, densely woven tents.
- An OPM colony of 400–1000 individuals produces 343–857 million setae within one season (Table 4).
- A single oak tree of 13 ± 4 m height and 40 ± 15 cm DBH (mean ± SD) can be infested by OPM with 28 ± 4 L(mean ± SE) of pupation tents during an outbreak culmination, for example. Regarding the mean tent volume, the potential contamination from such a tree with setae from larvae of the instars L3-L6 within one season would extend over the following ranges for loose vs. compact tents (Table 4):
  ∘ Mean (28 L): 9.6–24.0 billion (10^9^) setae;
  ∘ Lower limit of the 95% confidence interval (21 L): 7.2–18.0 billion setae;
  ∘ Upper limit of the 95% confidence interval (36 L): 12.9–30.9 billion setae.

Depending on OPM population density and oak tree size, trees can be infested with OPM pupation tents up to 121 L volume, which was the maximum during our studies of the OPM outbreak at Michaelisbruch, Germany, in 2016–2019 (for details, see Supplementary Data 2.3.4, Figure S4, Table S4; for geographic coordinates, see Supplementary Data 1, Table S1). In addition to the fresh tents from one season, pupation tents from previous years, not considered in this extrapolation, might enhance the setae contamination potential.

### 3.2. Airborne Setae Dispersion

#### 3.2.1. Close Range, Circular Dispersion

Air temperature and relative humidity measured on site during the experiment were 18 °C and 77%, respectively. Wind direction was mainly NW, N, NE for 9, 10, and 8 min with wind speeds of 0.6, 0.4, and 0.5 m/s, respectively. There was no precipitation.

##### Active and Passive Samplers:

At 3 m distance to the setae source, which consisted of approximately 1.4 billion (10^9^) setae (see below), the active sampler caught ca. 440 setae. This corresponds to a contamination of ca. 0.367 setae/L air, i.e., 367 setae/m^3^ air.

The two small passive samplers at 1.5 m AGL, which were installed next to the active sampler, caught ten setae on the horizontal and one seta on the vertical slide. Contrary to the twenty-two large passive samplers (see below), the small vertical passive sampler caught fewer setae than the horizontal. This was due to its turned positioning, i.e., not head-on to the air current.

**Figure 5:**
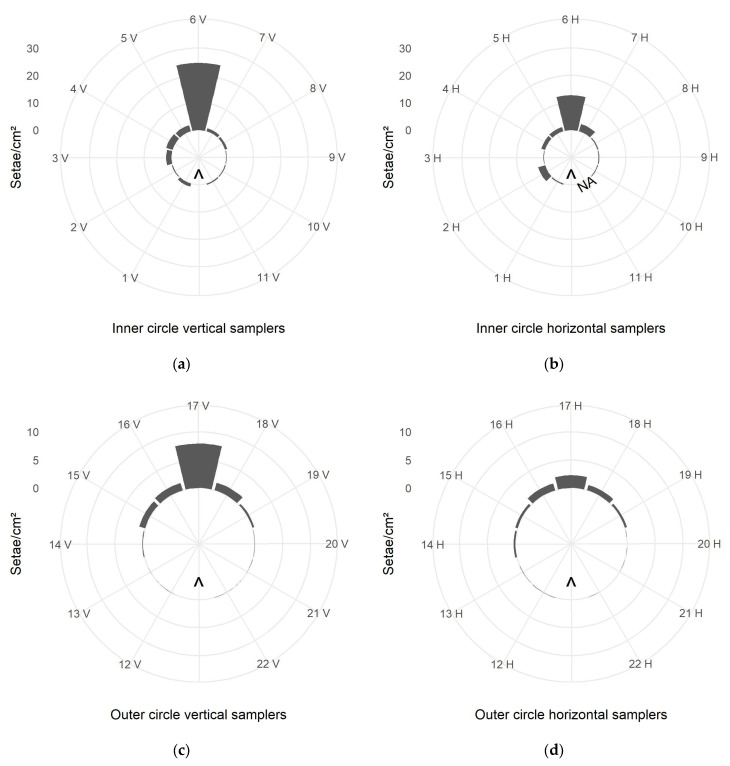
Number of setae per cm^2^ sampler, caught by the large passive samplers during the setae exposure simulation; (**a**,**b**)—samplers ID 1–11 in the inner circle at 1.5 m distance to the setae source (larger number of setae per cm^2^ than in the outer circle, see axis limit); (**c**,**d**)—samplers ID 12–22 in the outer circle at 3.0 m distance to the setae source; V—vertical; H—horizontal; samplers installed in 30° steps to each other around the setae source in the circle center; arrow—blower position and blowing direction; NA—data from sampler ID 11 H missing.

The evaluation of the large passive samplers in circular formation indicated turbulences in the setae-carrying air flow. Even at an angle of 150° to the direction of the air flow, setae were detected (Figure 5). But most setae were caught by the samplers in the immediate direction of the airstream, i.e., straight downwind, and the side spread of the setae declined markedly with increasing angle to the blowing direction.

The tendency of aerial setae drift in the east–southeast direction was discerned in both the inner and the outer sampler circles. As the blower orifice was directed southwards, this drift was caused by natural wind, which was not permanently parallel to the blowing direction. The samplers in the inner circle at 1.5 m distance to the setae source caught generally more setae. But the setae distribution across the different samplers was more irregular compared to the setae catch by the outer samplers at 3.0 m distance. The samplers installed at 0° to the air flow direction caught markedly larger numbers of setae than the samplers at higher angles to the air flow direction. This trend of airborne setae dispersion applied to the inner as well as the outer circle (Figure 5). However, in the outer circle, especially at the horizontal samplers, the sum of setae per cm^2^ sampler decreased more regularly and gradually in smaller steps with increasing angle to the direction of the air flow.

In general, higher vertical than horizontal setae deposition was observed in both the inner and the outer circle for those samplers standing in straight upwind direction, i.e., ID 6 and 17 (Figure 5).

##### Simulated Exposure of a Person (Dummy Experiment):

**Figure 6:**
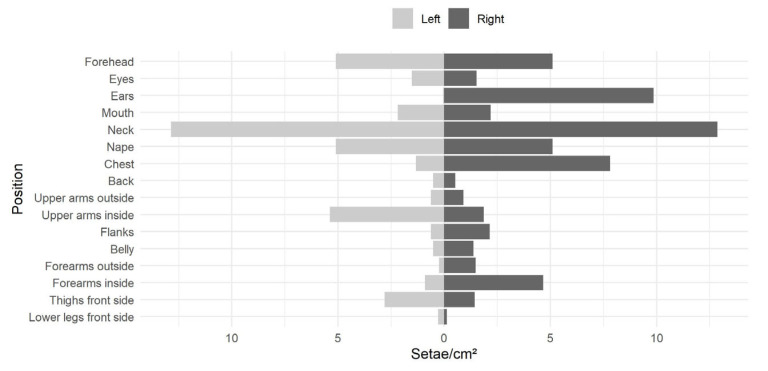
Setae contamination (number of setae per cm^2^ tape at the different tape positions) of the left and the right side of the dummy exposed at 2.5 m distance to the setae source in the simulation.

The analysis of the adhesive tapes on the dummy revealed an unequal setae distribution. The upper parts of the dummy, particularly the head, neck and nape, showed, on average, ca. threefold higher setae contamination than the rest of the body (Figure 6). Due to the positioning of the dummy in slight axial rotation to the air flow, the left ear caught less than 1% of the setae per cm^2^ than the right ear.

In total, 1387 setae were captured by all twenty-three tapes on the front side of the dummy, including the flanks and excluding the left side of the head. The total surface area of these tapes was 483 cm^2^. This resulted in ca. 2.9 setae/cm^2^ on average. Assuming a height of 1.80 m and a weight of 60 kg of a human with the same size as the dummy, the half-body surface area was 0.87 m^2^, according to the formula from Mosteller [97]. Hence, based on the extrapolation, a total number of ca. 24,900 setae might have contaminated the entire front surface of a human under the conditions given in this experiment.

On the upper back side of the dummy, i.e., the nape, back and left side of the head, 1.9 setae/cm^2^ were caught on average. On the upper front side including face and arms, 3.2 setae/cm^2^ were found. In comparison to the belly and the neck, the back and nape were contaminated by approximately half of the setae amount per cm^2^. Thus, the entire dummy, i.e., front and back, was contaminated with approximately 37,000 setae.

##### Setae Source Magnitude:

The natural OPM infestation level on the experimental site was low, as only 0.06 pupation tents per tree were recorded from 200 checked trees.

The used setae source of 10 pupation tents consisted of approximately 1760 individuals (exuviae excluded): 100 moths, 870 closed cocoons, 200 prepupae, 140 closed pupae without cocoons or from destroyed cocoons, and 450 empty or destroyed cocoons. The number of exuviae was estimated to be roughly at least 400. They originated mainly from the instar L5 and were not exactly countable owing to partial weathering and destruction. The setae source contained approximately 1.4 billion (10^9^) setae in total according to the mean setae amount per larva in the L5 and L6 instars (see Section 3.1). A sample of seven intact L5 exuviae was examined for the remaining setae amount, which resulted in 11% on average.

#### 3.2.2. Horizontal Setae Dispersion

Air temperature and relative humidity at the study site were 23 °C and 63%, respectively. There was no precipitation. The wind direction on site was mainly SE, S, SW for 7, 26, and 18 min, respectively. The wind speed was 2.8 m/s from SSE direction, measured at the official weather station Mattersburg, Austria (for geographic coordinates, see Supplementary Data 1, Table S2).

The active sampler at 5 m distance to the setae source caught 41 setae in total during the one-hour experiment. The pivoted small vertical passive samplers did not catch any setae, except one seta caught at 10 m distance (position ID 10).

**Figure 7:**
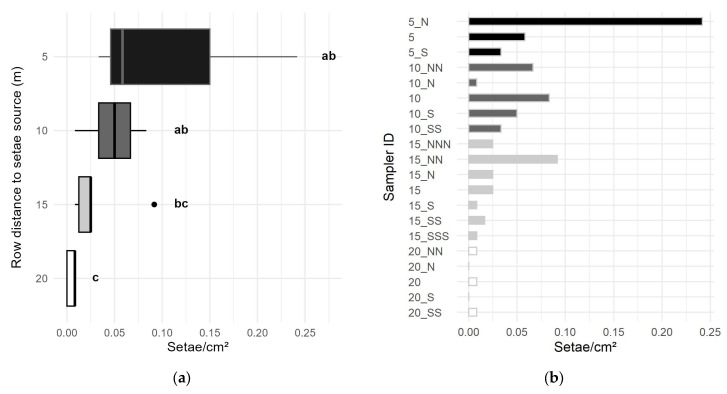
Number of setae per cm^2^ sampler, caught by large vertical passive samplers (size: 40 mm × 300 mm) in four rows at 5, 10, 15, and 20 m distance to the setae source in the setae exposure simulation. (**a**)—setae catch at the rows of samplers, statistical differences between 5 vs. 20 m and 10 vs. 20 m distance (indicated by compact letter display ab, bc and c; for statistics, see Supplementary Data 4.2.3); (**b**)—setae catch by the individual samplers at 5 m distance (ID 5_N, 5, 5_S), 10 m (from ID 10_NN to 10_SS), 15 m (from ID 15_NNN to 15_SSS) and 20 m distance (from ID 20_NN to 20_SS) to the setae source; N, S—sampler position in northward and southward deviation from the air flow of the blower, respectively.

The setae catch diminished with increasing distance to the setae source. The average number of setae per cm^2^ of sampler showed a decrease of approximately 50% from row to row between 5 m to 15 m distance (Figure 7). At 20 m distance, the setae catch decreased further to a proportion of 10% of the catch in the row at 15 m distance. Differences in the setae catch were only significant between the rows at 5 m vs. 20 m and 10 m vs. 20 m from the setae source (Figure 7a, for statistics, see Supplementary Data 4.2.3).

A northward drift of the setae was discerned, caused by natural wind from S and SW direction, i.e., in right angle to the air flow from the blower. Thus, the setae catch in southward deviation from the blowing direction was lower compared to that in northward deviation (Figure 7). However, no significant differences were ascertained between the rows in a diagonal direction (*p* = 0.279).

##### Setae Source Magnitude:

Compared to 2014, the natural OPM population density on site decreased further in 2015. Only two pupation tents were found at the ca. 50 oak trees bordering the opening.

During the experiment, at least 3000 living L6 larvae (roughly estimated) were used as setae source, corresponding to 40–50 L OPM larvae and molting tents (see Supplementary Data 4.1.3: Figure S8C). Approximately 3400 L5 exuviae were found in the mesh bags. The remaining setae number on 154 selected L5 exuviae was estimated at 12 ± 22% (mean ± SD).

## 4. Discussion

Marzano et al. [93] and Buist et al. [101] showed the lack of OPM-based knowledge and the need for further research on the identification of OPM-related health impacts to reach the required “preparedness for OPM”. Two main topics were considered in the present study, i.e., setae quantification and airborne setae exposure, which are essential for the management of OPM-related hazards.

### 4.1. Setae Quantification

The observed distribution of the setae-carrying mirrors across the larval body in the different instars corresponds to the findings of Scheidter [58] and Lamy et al. [64]. In accordance with Scheidter [58], some particularly large larvae carried setae on the mirrors of segments, which are usually free of setae and typically carry setae only in the following instar. The regular chronological sequence is characterized by setae-carrying fore-mirrors only in L6 and on segment 11 of L5, as well as the presence of setae-carrying dorso-lateral mirrors in L6. The deviation from this regular sequence was attributed to the vigorous constitution of these processionary larvae (cf. [58]).

The total setae-carrying area of the mirrors and the concomitant setae number depended on the larval body size. This was probably related to the nutritional status of the larvae and further environmental conditions because the larvae from the semi-field were generally smaller than those from the field (see also Supplementary Data 3.2 for the results of OPM rearing on an oak-free diet). The rearing conditions on oak twigs in water bottles in the semi-field were probably suboptimal, especially towards the end of the larval development. This was indicated by the increasing discrepancy in the total setae amount and in the setae-carrying mirror area per larva between larvae from the field and semi-field with rising larval age from L4 to L6. Accordingly, a larger quantity of setae is assumed for L3 in the field than was found in the samples from the semi-field in this study. To avoid underestimation of health hazards in the field, only data from field samples which were available for L4–L6 were used for the extrapolation of the setae production per OPM individual and colony.

Linked to the size of the larval body, the head capsule width was influenced by the environmental and nutritional conditions during larval development. The OPM larvae reared in the semi-field or on an oak-free diet in the laboratory had generally smaller head capsules than larvae of the same instars from the field (see Supplementary Data 3.2, [96]). The comparison of the within-instar head capsule width between distinct field origins revealed differences: Romania [96] vs. Upper Rhine Valley, Germany (own unpublished results). Hence, the larval instar of OPM should generally be determined by examination of the mirrors with a microscope rather than by the commonly used measurement of the head capsule (cf. [47]). This would also be recommendable in instar determination of other processionary and lepidopteran species, such as *Euproctis chrysorrhoea* [41], which carry setae on the abdominal mirrors, and which have a setae distribution characterized by an instar-specific sequence.

The setae sockets in the center of the mirrors of L4–L6 larvae had wider spaces to each other, and larger diameters, and might thus have carried larger setae than those at the edges of the mirrors. This impression of a definite, regular setae distribution within the mirrors was confirmed by SEM photos of OPM mirrors from which the setae were not mechanically removed [49]. Contrary to OPM, SEM photos of L5 larvae of PPM and *T. pinivora,* as well as L3–L8 larvae of *O. lunifer*, showed intermixed large and small setae sockets in the mirrors [49,63,76]. Large setae probably do not disperse as far and as rapidly as smaller setae due to their weight and size [49,67]. Therefore, during the period of OPM L4-L6, when large setae are formed, their dispersion is assumed in the immediate vicinity to the source. However, it is not yet known whether large setae pose greater health hazards than small ones (see also [49], cf. [36]).

While the total setae amount per OPM larva increases slightly from L3 to L5, it increases strongly at the last molting to L6. An OPM L5 larva carries only ca. 5–7% of the L6 setae amount. The upsurge in the setae amount per larva at the last instar also applies to PPM L5, *T. pinivora* L5, *E. chrysorrhoea* L5 and *O. lunifer* L8 (e.g., [41,58,76]. As the setae serve as protection against predatory vertebrates, the last instar needs considerably more setae than the preceding instars. The duration of OPM L6 development is usually two to three times longer than that of each of the prior instars [57,91]. Setae are released into the air by the larvae during locomotion outside their tents and form a protective “cloud” around the colonies and trees (own unpublished results; [49]). Thus, a sufficient releasable setae amount must be available on the larval body to ensure continuous protection throughout the whole last instar. In PPM, the setae especially protect the L5 larvae during the pre-pupation procession [77]. Furthermore, setae are needed at the end of the last instar to weave them into the cocoon or the pupal chamber for protection of the immobile pupae in OPM, PPM, *E. chrysorrhoea*, and *O. lunifer*, for example (own observations, [59,76]; cf. [36]).

In the present study, an improvement of the existing method for determining the setae quantity [60,61,76] was applied by standardized setae counting. This was conducted in each single mirror in squares of 25 µm × 25 µm across a grid of 100 µm × 100 µm to avoid arbitrariness and to ensure method repeatability and enable a comparison of the results. Furthermore, this allowed for covering different parts of the mirrors, as the setae density (setae number per area) varies within the mirrors (own observations: see above; cf. [76]).

The density of OPM setae ranged from ca. 31,000 to 62,000 setae/mm^2^ in the mirror sections of close-set sockets of L3 and L6 larvae, respectively. Similar setae densities were found in *O. lunifer*, ranging from ca. 31,000 to 93,000 setae/mm^2^, and 67,000 setae/mm^2^ on average in L3–L8 [76]. Like in *O. lunifer* [76], OPM setae density tended to increase with rising larval instar. The mean density of ca. 57.000 setae/mm^2^ of last instar OPM larvae (L6) from the field corresponded approximately to that of last-instar PPM larvae (L5), which is 60,000 setae/mm^2^ [60,61]. The setae densities and the total number of setae per larva, as well as the within-instar variability, were similar for L3–L5 of OPM and *O. lunifer* [76]. But compared to last-instar OPM larvae, the larvae of the last instars (L7 and L8) of *O. lunifer* carry more setae (ca. 1.4–2.5 million per larva), and the setae densities are higher in *O. lunifer* L7 and L8 larvae (ca. 67,000–93,000 setae/mm^2^) [76].

The present study gives the first report of the total setae number of OPM larvae, determined instar-specifically by a standardized measuring and counting procedure. The number of ca. 806,000 ± 13,000 (mean ± SD) setae per OPM L6 larva from the field was smaller than that reported for PPM L5, which carries ca. 1 million setae at a minimum [59,60], although both are the last instar of their respective species, and all mirrors are setae-studded [58,63]. Seldeslachts et al. [81] estimated a total of approximately 1 million setae per OPM L5–L6 instar larva. However, the instar was not exactly determined, and no standardized procedure was applied to ascertain the setae number. Instead, this number resulted from collecting setae from 200 larvae shaken in tubes for the analysis of the venom components [81].

Generally, high within-instar variability in the setae amount per larva was established in OPM in the present study and in *O. lunifer* by Perkins et al. [76], depending on the larval size. The variation coefficient of the OPM setae amount per larva in this study was 0.174 in L4, 0.124 in L5 and 0.016 in the L6 samples from the field from the Upper Rhine Valley, Germany. Further studies with a larger sample size and a broader range of sample origins should verify these results.

The setae number per OPM individual and per pupation tent was extrapolated, which enables a quick assessment of the setae contamination potential. OPM pupation tents often also contain exuviae of the preceding instars, especially of L5 ([58], own observations). The extrapolation, which indicated a whole-tree contamination with 9.6–24.0 billion (10^9^) setae, reflects a worst-case scenario and corresponds to an OPM outbreak situation with a mean volume of newly created pupation tents of 28 L per tree.

If such OPM population densities occur and the corresponding tent volumes per tree are found, OPM abundance has already been high in the previous year(s) and will be high in the following year(s), as OPM outbreaks last for several years without control measures (see Supplementary Data 2.3.4; e.g., [54] and references therein, [55]). Most setae remain bound to the exuviae or are spun into the cocoons, rather than being released into the air, as further studies on airborne OPM setae dispersion revealed (own unpublished results). Once infested by OPM, the setae accumulate on the trees and the surrounding vegetation and ground, forming a setae source persisting for years (own observations, [20,21,22]). This exacerbates the health hazards and leads to recurrent contaminations of humans and animals because the setae keep their harmful potential for years [21,59,74,75].

### 4.2. Airborne Setae Dispersion

A preliminary study was used to test the simulation of airborne setae exposure in the field. Based on the results (see Supplementary Data 4.3.2, Table S9), the experimental setup was adapted in the following experiments. As the natural OPM population density was at a latency level in both study years, there was no relevant release of setae from the trees neighboring the study site.

#### 4.2.1. Close Range, Circular Dispersion

##### Active and Passive Samplers:

As expected, the number of airborne setae diminished with increasing distance to the setae source. Moreover, the setae dispersed mainly parallel to the dominant air flow, as most setae were caught by the samplers placed straightly downwind, especially in the vertical samplers. This suggests an obstacle effect of these vertical samplers to the setae-dispersing air flow.

Apart from the predominantly straight-line setae dispersion, the arrangement of the samplers enabled the detection of turbulences up to 150° deviation from the generated air flow at distances of 1.5 m and 3.0 m from the source. Accordingly, the entire surroundings of a setae source, at least within this radius, are not free of airborne setae and must thus be considered a hazard zone. Nevertheless, it is essential to take the wind direction into account to minimize setae exposure if it cannot be completely avoided, e.g., in the case of occupational exposure.

The deposition (interception) of setae at the samplers in the outer circle was more regular than in the inner circle, corresponding to a Gaussian aerosol distribution ([67] and references therein, [102]). Based on these findings, the airborne setae concentration at 5–20 m distance from the source and up to 17–31° deviation from the main air flow direction was investigated in detail in the follow-up experiment in 2015.

##### Simulated Exposure of a Person (Dummy Experiment):

The total number of setae on the surface of the dummy located at 2.5 m distance to the setae source might deviate from the calculated approximately 37,000 setae, as the extrapolation was based only on the vertical interception of setae. Horizontal and oblique areas were not considered; for example, the top of the head, the shoulders and the inside and outside of the legs were not equipped with sampling tapes. Horizontal deposition was generally lower than vertical deposition of the setae, as shown by the catches of the passive samplers in the circle.

The occurrence of the highest setae contamination on the upper parts of the dummy was attributed to the installation of the setae source at 1.5 m AGL. Thus, the setae were spread straightly downwind, and only few setae had already descended to lower-body regions on their 2.5 m long way from the setae source to the dummy. The horizontal setae deposition was already measurable at 1.5 m downwind distance to the setae source at sampler ID 6, which was installed between the setae source and the dummy. However, this horizontal deposition of setae might have been caused by an obstacle effect of the sampler rather than by natural setae descending.

The dummy was exclusively exposed to airborne setae, the most frequent way of human contamination, although direct touching of setae represents another source of danger to humans [9,21,22,29]. Humans are particularly threatened due to the temporal coincidence of their outdoor activities frequently in light, short-sleeved clothing and the period of setae-carrying OPM larvae in late spring and early summer (own observations, cf. [22]). Due to its outdoor setup, this pilot study was considered a realistic simulation of a worst-case scenario of human health hazards. Compared to the dummy exposure, Olivieri et al. [29] observed a lower efficiency of the tape sampling method to determine the contamination level of tree fellers during harvesting of PPM-infested trees. This might be due to the following reasons: use of smaller tapes of 15 mm × 40 mm, shorter exposure time of 5–22 min, and smaller setae source of 20–300 larvae per tree compared to this study. Another reason for the lower efficiency might be that the sampling by tape impression test was carried out after the setae release, rather than during the setae release.

In general, the contamination hazards to people moving in the vicinity of infested trees are probably higher than the hazards estimated by use of a stationary dummy at 2.5 m distance from the setae source. The hazards are exacerbated by direct contact with the setae when handling contaminated material, e.g., during occupational exposure (e.g., [9,29], cf. [85]). Even at low infestation levels of 20–300 larvae per tree, Olivieri et al. [29] detected airborne contamination with 8.9 setae per working hour. During tree felling in the study, PPM setae were released from the tents containing L5 larvae and exuviae of the previous instars or from the vegetation [29].

#### 4.2.2. Horizontal Setae Dispersion

In the second experiment, the setup was modified so that the setae source consisted of living OPM larvae and their molting tents. Furthermore, the samplers were placed at larger distances up to 20 m from the setae source, and pivoted constructions allowing for the orientation of the passive samplers towards the wind direction were used.

Small-scale differences and turbulences in the airborne setae concentration up to 31° from the direction of the air flow were detected. The reason why only one seta was found on the pivoted vertical passive samplers might have been the limited impaction area of 20 cm^2^ per sampler, compared to 120 cm^2^ impaction area of the used static large vertical passive samplers.

The catch of larger setae amounts was linked to setae in clusters or attached to large particles, from which higher health hazards are assumed to emanate than from single setae. No setae clusters were found at 20 m distance in this experiment. Moreover, the findings by Olivieri et al. [29], who used the same distances in their studies, might suggest that distances of 15–20 m are outside the health hazard zone. The authors conducted their studies in the morning in late spring, under an overcast sky at 8–9 °C and 80–100% relative humidity. At 15–20 m distance from the setae source, contamination with airborne PPM setae was minimal when trees were felled, each infested with a PPM tent containing 20–300 L5 larvae [29].

In the experiment in 2015, a steady air flow was created by the blower. However, naturally occurring gusts striking the OPM larvae and tents used as setae source might have enhanced the release of setae clusters into the air. Studies in Brandenburg, Germany, during an OPM outbreak in 2018 and 2019 showed the natural aerial spread of setae clusters up to 450 m distance from the source (own unpublished results).

Despite the natural wind, the northward deflection of the airborne setae was not so distorting, as the differences in setae capture between the rows in the diagonal direction were not significant. Instead, the results clearly showed that the number of airborne setae depended on the distance of the sampler rows from the setae source. The dilution of the setae concentration in the atmosphere follows a Gaussian distribution: the larger the distance from the source, the higher the dispersal in horizontal and vertical directions [67] and references therein, [102].

#### 4.2.3. Summary: Airborne Setae Dispersion

The operation of sampling devices such as suction traps and the analysis of their setae catches are time-consuming and not practicable as part of continuous monitoring for annual hazard assessment of the airborne setae contamination (see also [29]). Therefore, the exposure simulations were conducted specifically under controlled and defined conditions to gain insights into possible worst-case scenarios of airborne setae contamination.

The exposure experiments aimed at a hazard simulation for seated or standing people and animals at ground level by placing the setae source at 1.5–2.0 m AGL and all passive samplers at ca. 1.3–1.5 m AGL or by using a dummy. In contrast, Fenk et al. [24] assumed a setae source at 20 m AGL for modeling of the airborne setae spread and for drawing conclusions on the spatio-temporal hazard range. This might correspond to OPM larvae in the upper periphery of the tree crown rather than to molting tents of L4 and L5 or pupation tents, which are usually built on lower parts of the trees. Or this might be more applicable to other processionary moth species which spin their tents in the upper tree crown, such as PPM. The higher the location of the setae source in a tree, the further away from the source the highest setae pollution occurs [67]. According to the model by Fenk [67] and Fenk et al. [24], the maximum setae concentration at a height of 3.8 m is reached at 562 m distance from the setae source at night (05:00 CEST) and at 174 m distance during the day (15:00 CEST). Modeling was based on a source of 104 setae/m^3^ air at a height of 20 m and weather conditions of a clear day. Under these conditions, the authors modeled an airborne setae pollution up to ca. 2 km downwind from the source at night and ca. 1 km during the day [24]. The dispersal range further depends on the length of the setae. Long PPM setae have an aerodynamic diameter of 13.6 ± 1.7 µm and short setae of 7.0 ± 1.0 µm mean ± SD [49]. From a source at 20 m height, long and short PPM setae travel up to 6.7 km and 25 km, respectively, during a day with a wind speed of 2 m/s [49].

Under natural conditions, setae release, dispersal and deposition are more variable due to the larger range of influencing factors, like humidity and air temperature (own unpublished results).

The findings obtained from the exposure simulation experiments are particularly valuable for the assessment of exposure to airborne setae in populated and urban areas. There, OPM frequently infests solitary trees, and turbulences are induced by wind deflection, acceleration or lowering by buildings. As indirect contact with airborne setae is the most common way of human exposure [9,21,29], the public should be warned, and workers, such as forestry staff, gardeners and landscapers, should be specially trained in dealing with the hazards and equipped to increase their occupational safety. This was also recommended by the authors mentioned above. Furthermore, this has been effectively implemented in the Netherlands by creating the “OPM Knowledge Centre” and the “OPM Knowledge Platform” [10]. Other examples of successful implementations are the “OPM Resource Hub” in the United Kingdom [103], and the free web application “PHENTHAUproc” in Germany [90], which is based on the OPM early-warning system [57].

## 5. Conclusions

The occurrence of OPM and the concomitant hazards, especially to human and animal health, are expected to persist and increase in the future. Oaks are becoming increasingly popular in urban and rural areas, as well as in forests, due to their supposed climatic adaptability and stability. The preferred host trees of OPM are solitary oaks or oaks in forest edges, i.e., areas which are highly frequented by humans during recreation. Thus, humans are more likely exposed to OPM setae.

The results of this study, together with a previous study [57], contribute to the handling and control of OPM infestations by providing factual knowledge. Here, insights into the OPM setae contamination potential are given. The extrapolation of the number of up to 900 million setae per single pupation tent of 1 L volume enables hazard assessment of possible contaminations by OPM-infested trees, especially regarding indirect exposure due to the airborne setae dispersal.

The results fill crucial knowledge gaps which challenge the development and realization of OPM adaptation strategies, compiled by Buist et al. [101]. Therefore, they should be incorporated into the respective information and warning campaigns to raise the awareness and risk perception of the public and of professional operators. This helps to form a basis for strategic decision-making. The facts generated ensure the implementation of appropriate measures in terms of integrated pest management and improve their general comprehension and acceptance. This is relevant to private and public land managers in regions where OPM is indigenous, but also where it was accidentally introduced or where it is immigrating to.

The findings presented here will be enhanced by the outcomes of our further studies on the spatio-temporal hazards of airborne setae dispersion under natural conditions, which will be published soon.

## Figures and Tables

**Table 1:** Wind speed measured at different positions of the experimental setup during the airborne setae exposure simulation performed to examine close range, circular setae dispersion; NA—not measured.

Measuring Position	Wind Speed (m/s) During the Experiment		
	16:15 (Start)	16:35	16:55
Blower orifice	12.50	NA	NA
Before mesh bag (setae source)	9.21	9.16	8.26
Behind mesh bag (setae source)	1.75	1.87	1.87
Samplers ID 3, 4, 7, 9, 14, 16, 18, 20	0.00	NA	NA
Sampler ID 5	0.87	1.10	0.86
Samplers ID 6, 17	0.70	NA	NA

**Table 2:** Setae quantity per OPM larva of the instars L3–L6, depending on the setae-carrying mirror area and the setae density; instar-specific means and standard deviation see Table 3; ID—consecutive numbering of the samples; origin—for geographic coordinates, see Supplementary Data 1, Table S1; semi-field—OPM reared on oak twigs in water bottles in a cage outdoors at FVA Freiburg; Freiburg field—Freiburg-St. Georgen; Mengen field—Schallstadt/Mengen; segments—setae-carrying larval segments; mirrors: f—fore, h—hind, d-l—dorso-lateral; ‡—additional setae-carrying mirrors deviating from the common distribution of setae-carrying mirrors on the larval body (only some samples); area—size of the setae-carrying mirror area; dense—close-set; wide—wide-spaced setae distribution in the mirrors; non-existent—no wide-spaced setae distribution in the mirrors of L3.

OPM Sample			Segments (Mirrors)	Area (mm^2^)			Setae/mm^2^		Setae/Larva		
Instar	ID	Origin		Dense	Wide	Total	Dense	Wide	Dense	Wide	Total
L3	1	Freiburg semi-field	11 (f, h)	0.074	Non-existent	0.074	40,145	Non-existent	2987	Non-existent	2987
	2			0.058		0.058	31,400		1808		1808
	3			0.041		0.041	49,120		2032		2032
	4			0.049		0.049	44,978		2201		2201
	5			0.040		0.040	56,640		2273		2273
L4	6	Freiburg field	10 (h), 11 (f, h)	0.159	0.040	0.198	47,171	24,793	7482	983	8465
	7			0.178	0.044	0.222	44,722	33,571	7957	1493	9451
	8			0.131	0.033	0.164	39,700	28,114	5207	922	6129
	9	Freiburg semi-field		0.102	0.025	0.127	38,400	27,200	3916	693	4610
	10			0.097	0.024	0.121	42,982	32,000	4156	774	4930
L5	11	Freiburg field	4-10 (h), 11 (f, h), [4-10 (f), 11 (d-l)] ‡	0.699	0.078	0.777	47,618	30,800	33,294	2395	35,690
	12			0.816	0.047	0.864	47,190	25,067	38,528	1189	39,718
	13			0.960	0.053	1.013	48,108	33,600	46,170	1793	47,963
	14	Lorraine field		1.202	0.121	1.323	47,086	28,036	56,600	3392	59,992
	15	Freiburg semi-field		0.404	0.060	0.464	54,479	46,488	22,017	2775	24,791
L6	16	Mengen field	4-10 (f, h), 11 (f, h, d-l), 12 (f), [6, 9, 10 (d-l)] ‡	12.419	1.215	13.635	60,804	39,408	755,155	47,889	803,044
	17			12.231	0.829	13.059	61,829	42,367	756,198	35,119	791,317
	18			16.869	0.569	17.438	47,536	36,158	801,896	20,567	822,463
	19	Freiburg semi-field		2.731	1.009	3.740	51,053	36,227	139,410	36,563	175,973
	20			7.906	0.275	8.182	45,492	30,375	359,674	8366	368,040

**Table 3:** Mean setae-carrying area, setae density and setae quantity per OPM larva of the instars L3–L6; individual data records see Table 2; SD—standard deviation; NA—no wide-spaced setae distribution in the mirrors of L3; sum of the minimum setae quantity per larva: 834,944; sum of the maximum setae quantity per larva: 882,863; *—sum of the means; %—percentage of the setae quantity per instar, related to the total setae quantity from L3 to L6.

OPM Sample			Area (mm^2^)		Setae/mm^2^				Setae/Larva		
					Dense		Wide				
Instar	ID	Origin	Mean	SD	Mean	SD	Mean	SD	Mean	SD	%
L3	1	Freiburg semi-field	0.052	0.013	44,457	8480	NA	NA	2260	397	0.3
	2										
	3										
	4										
	5										
L4	6	Freiburg field	0.195	0.024	43,865	3110	28,826	3619	8015	1393	0.9
	7										
	8										
L5	11	Freiburg field	0.885	0.097	47,639	375	29,822	3552	41,123	5108	4.8
	12										
	13										
L6	16	Mengen field	14.711	1.943	56,723	6510	39,311	2535	805,608	12,844	94.0
	17										
	18										
Total									857,006 *		100.0

**Table 4:** Approximate number of OPM individuals and setae per OPM colony (pupation tent) and per tree (extrapolation); number of setae according to the mean setae amount per larva from instar L3 to L6 (see Table 3); loose—less densely woven, compact—densely woven (see Supplementary Data 2.3.2); for tent size classification, see Supplementary Data 2.3.3; Ref.—reference tent volume of 1 L; tree of 13 m height and 40 cm DBH; CI—95% confidence interval.

Level	Size Class	Tent Volume (L)	Loose Tents		Compact Tents	
			Individuals	Setae (Million)	Individuals	Setae (Million)
OPM colony (tent)	Ref.	1.0	400	343	1000	857
	Small	0.2	80	69	200	171
	Medium	3.0	1200	1028	3000	2571
	Large	6.0	2400	2057	6000	5142
	Extra-large	12.0	4800	4114	12,000	10,284
Whole tree	CI lower limit	21.0	8400	7200	21,000	18,000
	Mean	28.0	11,200	9600	28,000	24,000
	CI upper limit	36.0	14,400	12,900	36,000	30,900

## Data Availability

The original contributions presented in this study are included in this article and the Supplementary Materials. Further inquiries can be directed to the corresponding author.

## Supplementary Materials

The following supporting information can be downloaded at: https://www.mdpi.com/article/10.3390/ijerph22091361/s1, Supplementary file: Technical and methodological details and background information. References [104,105,106,107,108,109,110] are cited in the Supplementary Materials.

## Abbreviations

The following abbreviations are used in this manuscript:

AS	Active sampler
AGL	Above ground level
BOKU	BOKU University (Universität für Bodenkultur Wien)
CEST	Central European Summer Time
d	Distance
d-l	Dorso-lateral mirror
DBH	Diameter at breast height
DWD	Deutscher Wetterdienst (German Meteorological Service)
f	Fore-mirror
FVA	Forest Research Institute Baden-Wuerttemberg (Forstliche Versuchs- und Forschungsanstalt Baden-Württemberg)
h	Hind-mirror
H	Horizontal
NA	Not available
n.r.	Not relevant
OPM	Oak processionary moth
PPM	Pine processionary moth
r	Radius
Ref.	Reference tent volume of 1 L
SD	Standard deviation
SE	Standard error
SEM	Scanning electron microscope
V	Vertical

## References

[1] Basso A., Negrisolo E., Zilli A., Battisti A., Cerretti P. (2017). A total evidence phylogeny for the processionary moths of the genus *Thaumetopoea* (Lepidoptera: Notodontidae: Thaumetopoeinae). Cladistics.

[2] Battisti A., Larsson S., Roques A. (2017). Processionary moths and associated urtication risk: Global change-driven effects. Annu. Rev. Entomol..

[3] Townsend M. (2008). Report on survey for oak processionary moth *Thaumetopoea processionea* (Linnaeus) (Lepidoptera: Thaumetopoeidae) (OPM) in London in 2007. Report to the Forestry Commission.

[4] Sands R.J. (2017). The Population Ecology of Oak Processionary Moth. Ph.D. Thesis.

[5] Stigter H., Romeijn G. (1992). *Thaumetopoea processionea* observed locally in large numbers in the Netherlands after more than a century (Lepidoptera: Thaumetopoeidae). Entomol. Ber..

[6] Tomiczek C., Krehan H. (2003). Zunehmende Probleme mit dem Eichenprozessionsspinner in Ostösterreich. Forstsch. Aktuell.

[7] Wagenhoff E., Delb H. (2011). Current status of *Thaumetopoea processionea* (L.) in South-Western Germany. Berichte Freibg. Forstl. Forsch..

[8] Groenen F., Meurisse N. (2012). Historical distribution of the oak processionary moth *Thaumetopoea processionea* in Europe suggests recolonization instead of expansion. Agric. For. Entomol..

[9] Vasseur P., Sinno-Tellier S., Rousselet J., Langrand J., Roques A., Bloch J., Labadie M. (2022). Human exposure to larvae of processionary moths in France: Study of symptomatic cases registered by the French poison control centres between 2012 and 2019. Clin. Toxicol..

[10] Buist Y., Bekker M., Vaandrager L., Koelen M., van Mierlo B. (2023). Strategies for public health adaptation to climate change in practice: Social learning in the Processionary Moth Knowledge Platform. Front. Public Health.

[11] Loock B., Lobinger G. (2016). Der Eichenprozessionsspinner—Situation in Bayern und praxisnahe Forschung im Waldschutz [The oak processionary moth—The situation in Bavaria and scientific approach in forest protection]. Jahrb. Baumpflege.

[12] Möller K., Heydeck P., Hielscher K., Pastowski F., Dahms C., Wenk M., Ebert P., Jacob C., Krüger A., Landesbetrieb Forst Brandenburg, Landeskompetenzzentrum Forst Eberswalde (2017). Blattfressende Insekten an Eiche und anderen Laubgehölzen. Waldschutzbericht 2017.

[13] Delb H., John R., Grüner J., Seitz G., Wußler J., Forstliche Versuchs- und Forschungsanstalt Baden-Württemberg (2018). Biotische Schaderreger an Laubbäumen. Waldzustandsbericht 2018 für Baden-Württemberg.

[14] Hlásny T., Mátyás C., Seidl R., Kulla L., Merganičová K., Trombik J., Dobor L., Barcza Z., Konôpka B. (2014). Climate change increases the drought risk in Central European forests: What are the options for adaptation?. For. J..

[15] Madrigal-González J., Ruiz-Benito P., Ratcliffe S., Rigling A., Wirth C., Zimmermann N.E., Zweifel R., Zavala M.A., Gil-Pelegrín E., Peguero-Pina J.J., Sancho-Knapik D. (2017). Competition drives oak species distribution and functioning in Europe: Implications under global change. Oaks Physiological Ecology. Exploring the Functional Diversity of Genus Quercus L..

[16] Mölder A., Meyer P., Nagel R.-V. (2019). Integrative management to sustain biodiversity and ecological continuity in Central European temperate oak (*Quercus robur*, *Q. petraea*) forests: An overview. For. Ecol. Manag..

[17] Jia P., Wang T., Van Vliet A.J.H., Skidmore A.K., van Aalst M. (2020). Worsening of tree-related public health issues under climate change. Nat. Plants.

[18] Kasper J., Weigel R., Walentowski H., Gröning A., Petritan A.M., Leuschner C. (2021). Climate warming-induced replacement of mesic beech by thermophilic oak forests will reduce the carbon storage potential in aboveground biomass and soil. Ann. For. Sci..

[19] Damestoy T., Jactel H., Belouard T., Schmuck H., Plomion C., Castagneyrol B. (2020). Tree species identity and forest composition affect the number of oak processionary moth captured in pheromone traps and the intensity of larval defoliation. Agric. For. Entomol..

[20] Weidner H. (1994). Belästigung von Menschen und Tieren durch die Raupen des Eichenprozessionsspinners *Thaumetopoea processionea* LINNAEUS 1758 (Lep., Thaumetopoeidae). Untere Havel—Naturkundliche Berichte.

[21] Maier H., Spiegel W., Kinaciyan T., Krehan H., Cabaj A., Schopf A., Hönigsmann H. (2003). The oak processionary caterpillar as the cause of an epidemic airborne disease: Survey and analysis. Br. J. Dermatol..

[22] Maier H., Spiegel W., Kinaciyan T., Hönigsmann H. (2004). Caterpillar dermatitis in two siblings due to the larvae of *Thaumetopoea processionea* L.; the oak processionary caterpillar. Dermatology.

[23] Fenk L., Vogel B., Horvath H. (2007). Dispersion of the bio-aerosol produced by the oak processionary moth. Aerobiologia.

[24] Vega J.M., Moneo I., García-Ortiz J.C., González-Muñoz M., Ruiz C., Rodríguez-Mahillo A.I., Roques A., Vega J. (2015). IgE sensitization to *Thaumetopoea pityocampa*: Diagnostic utility of a setae extract, clinical picture and associated risk factors. Int. Arch. Allergy Immunol..

[25] Schröder J., Wenning A., Hentschel R., Möller K. (2016). Rückkehr eines Provokateurs: Was steuert die Ausbreitungsdynamik des Eichenprozessionsspinners in Brandenburg?—Ergebnisse aus dem Waldklimafonds-Projekt “WAHYKLAS”. Eberswalder Forstl. Schriftenreihe.

[26] Barthod C., Zmirou-Navier D. (2018). Un tournant dans la prise en compte des arbres et des forêts en santé publique. Rev. For. Française.

[27] Thibaudon M., Besancenot J.-P. (2018). Forêts et allergies. Rev. For. Française.

[28] Vornholt C.-P. (2020). Verpflichtung zur Beseitigung von EPS-Gespinstnestern. AFZ-Der Wald.

[29] Olivieri M., Ludovico E., Battisti A. (2023). Occupational exposure of forest workers to the urticating setae of the pine processionary moth *Thaumetopoea pityocampa*. Int. J. Environ. Res. Public Health.

[30] Crandall-Stotler B.J., Bartholomew-Began S.E. (2007). Morphology of mosses (phylum Bryophyta). Flora N. Am. N. Mex..

[31] Antonín V., Ryoo R., Ka K.H. (2014). Marasmioid and gymnopoid fungi of the Republic of Korea. 7. Gymnopus sect. Androsacei. Mycol. Prog..

[32] Owari Y., Nakamura F., Oaki Y., Tsuda H., Shimode S., Imai H. (2022). Ultrastructure of setae of a planktonic diatom, *Chaetoceros coarctatus*. Sci. Rep..

[33] Crouau Y. (1997). Comparison of crustacean and insect mechanoreceptive setae. Int. J. Insect Morphol. Embryol..

[34] Christian A., Karg W. (2008). A revised setal nomenclature based on ontogenetic and phylogenetic characters and universally applicable to the idiosoma of Gamasina (Acari, Parasitiformes). Soil Org..

[35] Winterton S.L. (2009). Scales and setae. Encyclopedia of Insects.

[36] Bertani R., Guadanucci J.P.L. (2013). Morphology, evolution and usage of urticating setae by tarantulas (Araneae: Theraphosidae). Zoologia.

[37] Shen X., Marcos Fu H.C. (2020). How the bending mechanics of setae modulate hydrodynamic sensing in copepods. Limnol. Oceanogr..

[38] Garner A.M., Russell A.P. (2021). Revisiting the classification of squamate adhesive setae: Historical, morphological and functional perspectives. R. Soc. Open Sci..

[39] Niederegger S., Gorb S., Jiao Y. (2002). Contact behaviour of tenent setae in attachment pads of the blowfly *Calliphora vicina* (Diptera, Calliphoridae). J. Comp. Physiol. A.

[40] Battisti A., Holm G., Fagrell B., Larsson S. (2011). Urticating hairs in arthropods: Their nature and medical significance. Annu. Rev. Entomol..

[41] Werno J., Lamy M. (1994). Daily cycles for emission of urticating hairs from the pine processionary caterpillar (*Thaumetopoea pityocampa* S.) and the brown tail moth (*Euproctis chrysorrhoea* L.) (Lepidoptera) in laboratory conditions. Aerobiologia.

[42] Berardi L., Battisti A., Negrisolo E. (2015). The allergenic protein Tha p 2 of processionary moths of the genus *Thaumetopoea* (Thaumetopoeinae, Notodontidae, Lepidoptera): Characterization and evolution. Gene.

[43] Battisti A., Walker A.A., Uemura M., Zalucki M.P., Brinquin A.-S., Caparros-Megidos R., Gachet E., Kerdelhué C., Desneux N. (2024). Look but do not touch: The occurrence of venomous species across Lepidoptera. Entomol. Gen..

[44] Gäbler H. (1954). Die Prozessionsspinner.

[45] Démolin G. (1963). Les “miroirs” urticants de la processionnaire du pin (*Thaumetopoea pityocampa* Schiff.). Rev. Zool. Agric. Appliquée.

[46] Plugaru S.G. (1968). The biology of the oak processionary moth in Moldavia. Vrednaja Polezn. Fauna Bespozvononych Mold..

[47] Lamy M. (1990). Contact dermatitis (erucism) produced by processionary caterpillars (Genus *Thaumetopoea*). J. Appl. Entomol..

[48] Walker A.A., Perkins L.E., Battisti A., Zalucki M.P., King G.F. (2023). Proteome of urticating setae of *Ochrogaster lunifer*, a processionary caterpillar of medical and veterinary importance, including primary structures of putative toxins. Proteomics.

[49] Petrucco Toffolo E., Zovi D., Perin C., Paolucci P., Roques A., Battisti A., Horvath H. (2014). Size and dispersion of urticating setae in three species of processionary moths. Integr. Zool..

[50] Barbaro L., Battisti A. (2011). Birds as predators of the pine processionary moth (Lepidoptera: Notodontidae). Biol. Control.

[51] Sobczyk T., Bundesamt für Naturschutz (2014). Der Eichenprozessionsspinner in Deutschland: Historie—Biologie—Gefahren—Bekämpfung. BfN-Skripten, 365.

[52] Mühlfeit M., Rumpf S., Rohde M., Plašil P., Sennhenn-Reulen H., Nordwestdeutsche Forstliche Versuchsanstalt (2021). Erhebung Wichtiger Phänologischer Daten und Korrespondierender Populationsdichten des Eichenprozessionsspinners (*Thaumetopoea processionea* L.) Sowie Untersuchung des Einflusses Seiner Natürlichen Gegenspieler Unter Unterschiedlichen Klimatischen Bedingungen in Verschiedenen Regionen Deutschlands im Rahmen des Verbundvorhabens “Modellgestützte Gefährdungsabschätzung des Eichenprozessionsspinners im Klimawandel (ModEPSKlim)”. Schlussbericht zum Teilprojekt 2A..

[53] Pascual J.A. (1988). Biología de la Procesionaria del roble (*Thaumetopoea processionea* L.) (Lep. Thaumetopoeidae) en el centro-oeste de la Península Ibérica. Boletín de sanidad vegetal. Plagas.

[54] Wagenhoff E., Veit H. (2011). Five years of continuous *Thaumetopoea processionea* monitoring: Tracing population dynamics in an arable landscape of South-Western Germany. Gesunde Pflanz..

[55] Klapwijk M.J., Csóka G., Hirka A., Björkman C. (2013). Forest insects and climate change: Long-term trends in herbivore damage. Ecol. Evol..

[56] Wagenhoff E., Wagenhoff A., Blum R., Veit H., Zapf D., Delb H. (2014). Does the prediction of the time of egg hatch of *Thaumetopoea processionea* (Lepidoptera: Notodontidae) using a frost day/temperature sum model provide evidence of an increasing temporal mismatch between the time of egg hatch and that of budburst of *Quercus robur* due to recent global warming?. Eur. J. Entomol..

[57] Halbig P., Stelzer A.-S., Baier P., Pennerstorfer J., Delb H., Schopf A. (2024). PHENTHAUproc-An early warning and decision support system for hazard assessment and control of oak processionary moth (*Thaumetopoea processionea*). For. Ecol. Manag..

[58] Scheidter F. (1934). Auftreten der “Gifthaare” bei den Prozessionsspinnerraupen in den einzelnen Stadien. Pflanzenkrankh. Pflanzenschutz.

[59] Lamy M. (1990). Chenilles et papillons urticants: Une “pollution” méconnue. La Rech..

[60] Ducombs G., Lamy M., Bergaud J.J., Tamisier J.M., Gervais C., Texier L. (1979). La chenille processionnaire. Enquête épidémiologique. Ann. Dermatol. Vénéréologie.

[61] Lamy M., Ducombs M., Pastureaud M.-H., Vincendeau P. (1982). Productions tégumentaires de la processionnaire du pin (*Thaumetopoea pityocampa* Schiff.) (Lépidoptères). Appar. Urticant Appar. Ponte. Bull. Société Zool. Fr..

[62] Stigter H., Geraedts W.H.J.M., Spijkers H.C.P. (1997). *Thaumetopoea processionea* in the Netherlands: Present status and management perspectives (Lepidoptera: Notodontidae). Proc. Sect. Exp. Appl. Entomol. Neth. Entomol. Soc..

[63] Battisti A., Paolucci P., Petrucco Toffolo E., Roques A., Roques A. (2015). Comparative structure of the urticating apparatus in processionary moths. Processionary Moths and Climate Change: An Update.

[64] Lamy M., Novak F., Duboscq M.F., Ducombs G., Maleville J. (1988). La chenille processionnaire du chêne (*Thaumetopoea processionea* L.) et l’homme: Appareil urticant et mode d’action. Ann. Dermatol. Vénéréologie.

[65] Vega J., Vega J.M., Moneo I., Armentia A., Caballero M.L., Miranda A. (2004). Occupational immunologic contact urticaria from pine processionary caterpillar (*Thaumetopoea pityocampa*): Experience in 30 cases. Contact Dermat..

[66] Fagrell B., Jörneskog G., Salomonsson A.C., Larsson S., Holm G.R. (2008). Skin reactions induced by experimental exposure to setae from larvae of the northern pine processionary moth (*Thaumetopoea pinivora*). Contact Dermat..

[67] Fenk L. (2005). Die Atmosphärische Ausbreitung der Brennhärchen des Eichenprozessionsspinners. Diploma Thesis.

[68] Rodríguez-Mahillo A.I., González-Muñoz M., Vega J.M., López J.A., Yart A., Kerdelhué C., Camafeita E., Garcia Ortiz J.C., Vogel H., Petrucco Toffolo E. (2012). Setae from the pine processionary moth (*Thaumetopoea pityocampa*) contain several relevant allergens. Contact Dermat..

[69] Seldeslachts A., Maurstad M.F., Øyen J.P., Undheim E.A.B., Peigneur S., Tytgat J. (2024). Exploring oak processionary caterpillar induced lepidopterism (Part 1): Unveiling molecular insights through transcriptomics and proteomics. Cell. Mol. Life Sci..

[70] Lamy M., Pastureaud M.H., Novak F., Ducombs G., Vincedeau P., Maleville J., Texier L. (1986). Thaumetopoein: An urticating protein from the hairs and integument of the pine processionary caterpillar (*Thaumetopoea pityocampa* Schiff., Lepidoptera, Thaumetopoeidae). Toxicon.

[71] Novak F., Pelissou V., Lamy M. (1987). Comparative morphological, anatomical and biochemical studies of the urticating apparatus and urticating hairs of some Lepidoptera: *Thaumetopoea pityocampa* Schiff., *Th. processionea* L. (Lepidoptera, Thaumetopoeidae) and *Hylesia metabus* Cramer (Lepidoptera, Saturniidae). Comp. Biochem. Physiol. Part A.

[72] Moneo I., Vega J.M., Caballero M.L., Vega J., Alday E. (2003). Isolation and characterization of Tha p 1, a major allergen from the pine processionary caterpillar *Thaumetopoea pityocampa*. Allergy.

[73] Rodríguez-Mahillo A.I., Carballeda-Sangiao N., Vega J.M., García-Ortiz J.C., Roques A., Moneo I., González-Muñoz M. (2015). Diagnostic use of recombinant Tha p 2 in the allergy to *Thaumetopoea pityocampa*. Allergy.

[74] Hase A. (1939). Über den Pinienprozessionsspinner und über die Gefährlichkeit seiner Raupenhaare. (*Thaumetopoea pityocampa* Schiff.). Anz. Für Schädlingskunde.

[75] Maronna A., Stache H., Sticherling M. (2008). Lepidopterism—Oak processionary caterpillar dermatitis: Appearance after indirect out-of-season contact. J. Dtsch. Dermatol. Ges..

[76] Perkins L.E., Zalucki M.P., Perkins N.R., Cawdell-Smith A.J., Todhunter K.H., Bryden W.L., Cribb B.W. (2016). The urticating setae of *Ochrogaster lunifer*, an Australian processionary caterpillar of veterinary importance. Med. Vet. Entomol..

[77] Uemura M., Perkins L.E., Zalucki M.P., Battisti A. (2020). Movement behaviour of two social urticating caterpillars in opposite hemispheres. Mov. Ecol..

[78] Werno J., Lamy M. (1990). Atmospheric pollution of animal origin: The urticating hairs of the processionary caterpillar (*Thaumetopoea pityocampa* Schiff.) (Insects, Lepidoptera). Comptes Rendus L’académie Sciences. Série III Sci. Vie.

[79] Forkel S., Mörlein J., Sulk M., Beutner C., Rohe W., Schön M.P., Geier J., Buhl T. (2021). Work-related hazards due to oak processionary moths: A pilot survey on medical symptoms. J. Eur. Acad. Dermatol. Venereol..

[80] Tan M.K.H., Jalink M.B., Sint Jago N.F.M., Ho L., Arnold van Vliet J.H., Das T., de Faber J.T.H.N., Wisse R.P.L. (2021). Ocular complications of oak processionary caterpillar setae in the Netherlands; case series, literature overview, national survey and treatment advice. Acta Ophthalmol..

[81] Seldeslachts A., Undheim E.A.B., Vriens J., Tytgat J., Peigneur S. (2024). Exploring oak processionary caterpillar induced lepidopterism (part 2): Ex vivo bio-assays unmask the role of TRPV1. Cell. Mol. Life Sci..

[82] Rebollo S., Moneo I., Vega J.M., Herrera I., Caballero M.L. (2002). Pine processionary caterpillar allergenicity increases during larval development. Int. Arch. Allergy Immunol..

[83] Santos-Magadán S., González de Olano D., Bartolomé-Zavala B., Trujillo-Trujillo M., Meléndez-Baltanás A., González-Mancebo E. (2009). Adverse reactions to the processionary caterpillar: Irritant or allergic mechanism?. Contact Dermat..

[84] Nikolov G., Kandova Y., Petrunov B., Mirchev P., Georgiev G. (2020). Skin reactions to allergens from processionary caterpillars (genus *Thaumetopoea*). Probl. Infect. Parasit. Dis..

[85] Renström A. (2002). Exposure to airborne allergens: A review of sampling methods. J. Environ. Monit..

[86] Ehrnsperger A. (2011). Fallstudie über die Emission von Gifthärchen aus Waldbeständen mit Eichenprozessionsspinner-Befall. Bachelor’s Thesis.

[87] Hirst J.M. (1952). An automatic volumetric spore trap. Ann. Appl. Biol..

[88] Burkard Manufacturing Co., Ltd. (2025). Seven-day recording volumetric spore trap. https://burkard.co.uk/product/7-day-recording-volumetric-spore-trap/.

[89] Roques L., Soubeyrand S., Garnier J., Battisti A., Robinet C., Roques A., Rousselet J. (2013). URTIRISK Software. https://biosp.mathnum.inrae.fr/URTIRISK.

[90] DWD—Deutscher Wetterdienst (2025). Eichenprozessionsspinner-Frühwarnsystem. https://www.dwd.de/eichenprozessionsspinner.

[91] Delb H., Halbig P., Seitz G., Wagenhoff E. (2019). Der Eichenprozessionsspinner als Profiteur des Klimawandels: Müssen Baum und Mensch mit dieser Gefahr leben?. Jahrb. Baumpflege.

[92] Klapwijk M.J., Walter J.A., Hirka A., Csóka G., Björkman C., Liebhold A.M. (2018). Transient synchrony among populations of five foliage-feeding Lepidoptera. J. Anim. Ecol..

[93] Marzano M., Ambrose-Oji B., Hall C., Moseley D. (2020). Pests in the city: Managing public health risks and social values in response to oak processionary moth (*Thaumetopoea processionea*) in the United Kingdom. Forests.

[94] EPPO, European and Mediterranean Plant Protection Organization (2022). EPPO Standard on phytosanitary procedures. EPPO Bull..

[95] Tomlinson I., Potter C., Bayliss H. (2015). Managing tree pests and diseases in urban settings: The case of oak processionary moth in London, 2006–2012. Urban For. Urban Green..

[96] Dissescu G., Ceianu I. (1968). Cercetari Asupra Bioecologiei Omizii Procesionare a Stejarului (*Thaumetopoea processionea* L.).

[97] Mosteller R.D. (1987). Simplified calculation of body-surface area. N. Engl. J. Med..

[98] Mangiafico S.S. (2025). Rcompanion: Functions to Support Extension Education Program Evaluation, Version 2.5.0..

[99] Ogle D.H., Doll J.C., Wheeler A.P., Dinno A. (2025). FSA: Simple Fisheries Stock Assessment Methods, Version 0.10.0..

[100] R Core Team (2025). R: A Language and Environment for Statistical Computing.

[101] Buist Y., Bekker M., Vaandrager L., Koelen M. (2021). Understanding public health adaptation to climate change: An explorative study on the development of adaptation strategies relating to the oak processionary moth in the Netherlands. Int. J. Environ. Res. Public Health.

[102] Turner D.B. (1970). Workbook of Atmospheric Dispersion Estimates.

[103] Forestry Commission OPM Resource Hub. Department for Environment 2025, Food and Rural Affairs..

[104] Straw N.A., Hoppit A., Branson J. (2019). The relationship between pheromone trap catch and local population density of the oak processionary moth *Thaumetopoea processionea* (Lepidoptera: Thaumetopoeidae). Agric. For. Entomol..

[105] Aschmann V., Lobinger G. (2023). Adaptives Risikomanagement in trockenheitsgefährdeten Eichen- und Kiefernwäldern mit Hilfe integrativer Bewertung und angepasster Schadschwellen (ARTEMIS): Waldschutzrisikomanagement mit variablen Schadschwellen für ausgewählte Bestandesschädlinge der Eiche in Süddeutschland. Schlussbericht zum Teilvorhaben 4. Bayerische Landesanstalt für Wald und Forstwirtschaft, Ed. https://projekte.fnr.de/index.php?id=18415&fkz=22020618.

[106] Williams D.T., Straw N., Townsend M., Wilkinson A.S., Mullins A. (2013). Monitoring oak processionary moth *Thaumetopoea processionea* L. using pheromone traps: The influence of pheromone lure source, trap design and height above the ground on capture rates. Agric. For. Entomol..

[107] Roversi P.F. (2008). Aerial spraying of *Bacillus thuringiensis* var. *kurstaki* for the control of *Thaumetopoea processionea* in Turkey oak woods. Phytoparasitica.

[108] Habermann M. (2012). Abschätzung von Schad- und Bekämpfungsschwellen beim Eichenprozessionsspinner. AFZ-Der Wald.

[109] Keena M.A., Vandel A., Pultar O. (2010). Phenology of *Lymantria monacha* (Lepidoptera: Lymantriidae) laboratory reared on spruce foliage or a newly developed artificial diet. Ann. Entomol. Soc. Am..

[110] Bergomaz R., Boppré M. (1986). A simple instant diet for rearing Arctiidae and other moths. J. Lepid. Soc..

