# Supplementary material for: Quantification of Urticating Setae of Oak Processionary Moth (Thaumetopoea processionea) and Exposure Hazards
Source: Int J Environ Res Public Health. 2025 Aug 29;22(9):1361. doi: 10.3390/ijerph22091361 (PMC12469864; doi:10.3390/ijerph22091361)
Supplement: Supplementary file 1 [file ijerph-22-01361-s001.zip › ijerph-3640809-supplementary.pdf]

Supplementary file:

Technical and methodological details and background information

Quantification of urticating setae of oak processionary moth (*Thaumetopoea processionea*) and exposure hazards

Contents

1 Supplementary data 1 – Geographic coordinates and climate data 1

2 Supplementary data 2 – Setae quantification 3

2.1 Collection of the semi-field OPM samples 3

2.2 Mirror area measurement and setae counting 3

2.2.1 Methods 3

2.2.2 Results: Irregular distribution of setae-carrying mirrors..................................... 3

2.3 OPM pupation tent volume.. 6

2.3.1 Setae in pupation tents.. 6

2.3.2 Volume range and number of individuals... 7

2.3.3 Pupation tent size classification... 9

2.3.4 Whole-tree tent volume 10

3 Supplementary data 3 – OPM laboratory-rearing on oak-free diets........................12

3.1 Methods 12

3.2 Results...13

3.3 Discussion 15

4 Supplementary data 4 – Airborne setae dispersion 16

4.1 Technical equipment and study setup 16

4.1.1 Active samplers..16

4.1.2 Pivoted passive samplers..16

4.1.3 Sampler arrangement 16

4.1.4 Weather station 17

4.2 Analysis 20

4.2.1 Sampler screening 20

4.2.2 Setae source magnitude 20

4.2.3 Statistical analysis...21

4.3 Preliminary study 22

4.3.1 Methods 22

4.3.2 Results..23

4.3.3 Discussion...24

4.4 Experimental limitations 25

1 Supplementary data 1—Geographic coordinates and climate data

Table S1. Key figures of the OPM study sites.

Approximate coordinates in decimal degrees (WGS 84), altitude given by the operator of the reference weather station and rounded to the nearest 10 m; a.s.l.—above sea level; Abun—OPM abundance during the study years: L—latency, N—noticeable infestation, O—outbreak; n. r.—not relevant; Temp—mean air temperature; Precip—mean annual precipitation sum; FVA—Forest Research Institute Baden-Wuerttemberg; DWD—Deutscher Wetterdienst; LTZ—Landwirtschaftliches Technologiezentrum Augustenberg; GeoSphere Austria—Bundesanstalt für Geologie, Geophysik, Klimatologie und Meteorologie; Météociel—Meteociel.fr (Observations, Prévisions, Modèles en Temps réel); NA—data not available.

Site	Country	Federal state	Region	Abun	Latitude (°N)	Longitude (°E)	Altitude (m a.s.l.)	Weather (long-term mean)			Reference weather station	Weather (recent mean)		
								Temp (°C)	Precip (mm)	Reference period		Temp (°C)	Precip (mm)	Reference period
Schallstadt/ Mengen	Germany	Baden-Wuerttemberg	Upper Rhine Valley	N, L	47.955388	7.735058	230	10.8	759.6	1995-2018	LTZ: Mengen	11.3	640.8	2011-2018
Freiburg-St. Georgen				L	47.983929	7.788740	230	10.8	759.6	1995-2018		11.3	640.8	2011-2018
Freiburg FVA				n. r.	47.976021	7.844211	300	10.8	759.6	1995-2018		11.3	640.8	2011-2018
Grezhausen				O, L	47.957627	7.632330	200	10.2	966.0	1981-2010	DWD: Müllheim	10.7	798.7	2011-2018 (2013 NA)
Michaelis-bruch		Brandenburg	Ostprignitz-Ruppin	O	52.758146	12.515425	30	9.2	574.0	1981-2010	DWD: Kyritz	9.8	587	2011-2018

Table S1 (continued)

Site	Country	Federal state	Region	Abun	Latitude (°N)	Longitude (°E)	Altitude (m a.s.l.)	Weather (long-term mean)			Reference weather station	Weather (recent mean)		
								Temp (°C)	Precip (mm)	Reference period		Temp (°C)	Precip (mm)	Reference period
Klingenbach	Austria	Burgen-land	Neusiedlersee-Hügelland	L	47.763517	16.557534	230	10.9	678.7	2008-2018	GeoSphere Austria: Mattersburg	11.0	650.5	2011-2018
Lainzer Tiergarten		Vienna	Hietzing	L	48.169506	16.254112	270	10.3	786.6	1998-2018	GeoSphere Austria: Vienna (Mariabrunn)	10.7	757.6	2011-2018
Pötzleinsdorf			Währing	L	48.242246	16.300353	290	10.2	640.7	1955-2018	GeoSphere Austria: Vienna (Hohe Warte)	11.6	645.3	2011-2018
Fénétrange	France	Lorraine	Moselle	O	48.880522	7.002497	260	NA	NA	NA	Météociel: Rémering-lès-Puttelange	12.3	1011.1	2018

Table S2. Geographic position of the reference weather stations.

Coordinates in decimal degrees (WGS 84); LTZ—Landwirtschaftliches Technologiezentrum Augustenberg, DWD—Deutscher Wetterdienst; GeoSphere Austria—Bundesanstalt für Geologie, Geophysik, Klimatologie und Meteorologie; station ID and altitude given by the operator.

Site	Country	Operator	Station ID	Latitude (°N)	Longitude (°E)	Altitude (m a.s.l)
Mengen	Germany	LTZ	20	47.950572	7.714836	212
Freiburg		DWD	1443	48.023276	7.834441	236
Mattersburg	Austria	GeoSphere Austria	11189	47.745000	16.389167	284

2 Supplementary data 2—Setae quantification

2.1 Collection of the semi-field OPM samples

The semi-field samples were obtained from OPM larvae reared on oak twigs put in water bottles in an outdoor cage at the Forest Research Institute Baden-Wuerttemberg (FVA) in Freiburg. The oak twigs were obtained from *Quercus robur* trees at Freiburg-St. Georgen and Grezhausen, Germany (for geographic coordinates, see Table S1).

2.2 Mirror area measurement and setae counting

2.2.1 Methods

In the mirrors of the L4 samples and the sample ID 20 of L6, the distribution of the sections of wide-spaced setae sockets was irregular and unjoined. Therefore, the area size of these sections was not measured, but their percentage of the total setae-carrying area of the mirrors was estimated.

For each mirror, i.e., fore, hind, left and right, if present on the respective body segment, four counting squares of 25 µm × 25 µm each were examined (Figure S1).

As the socket density varied in different sections of the mirror, the squares were arranged at 100 µm distance to each other, either in line transect or grid. In case not all sockets were visible in a square on the SEM-photo, e.g., because of cover by true hairs, the square was placed at the next 100 µm distant point of the line transect or corner of the grid. If the mirror was too small, the number of its counting squares was reduced from 4 to 1-3, and the distance between the squares was reduced while keeping an even distribution across the mirror.

At the periphery of each counting square, only those setae sockets at the upper and the left edge were counted. Counting squares were not used for the dorso-lateral mirrors. Visual examination revealed only close-set setae sockets in these mirrors.

2.2.2 Results: Irregular distribution of setae-carrying mirrors

The regular distribution of setae-carrying areas in the larval mirrors of OPM and other processionary species was described by Scheidter [58] and Lamy et al. [64].

Interestingly, in this study, the fore-mirrors of the segments 4-10 of the L5 larvae were not always free of setae, and some sections carried few setae (ca. 1-30 setae; see article Section 3.1.: Figure 4a). These sections seemed randomly spread across the fore-mirrors, and they were not connected to each other.

Regularly, setae-carrying dorso-lateral mirrors only occur in segment 11 of L6 larvae [58]. However, the L5 and L6 larvae examined in this study formed setae-carrying dorso-lateral mirrors (see article Section 3.1.: Figure 4b). For example, segment 11 of the L6 larvae always had and other segments from the 6th onwards occasionally had setae-carrying dorso-lateral mirrors. But compared to segment 11, the dorso-lateral mirrors of the segments 6-10 were very small and carried ca. 30-200 setae each. They occurred sporadically only on one body side, either left or right.

Furthermore, the setae-carrying sections within the mirrors were not necessarily connected. In some samples, scattered small areas occurred besides the “main” (large) setae-carrying mirror area (see article Section 3.1.: Figure 4e).

Consistently across all samples of the different instars, true hairs were found singly and sparsely within the mirrors (see article Section 1.: Figure 1c and Section 3.1.: Figure 4c). They were firmly fixed to the integument and not mechanically removable with forceps. Their setae-detaching function by setae pulling and levering during mirror closing and opening when the larvae move was described by Battisti et al. [63] and references therein (see also Weidner [20]).

Depending on the instar, the setae distribution within the mirrors varied. In contrast to L3, the examined L5, L6 and some L4 samples had mirror sections with wide-spaced setae distribution. These sections were characterized by approximately double the space between the setae sockets, compared to the rest of the respective mirror which had close-set setae sockets (see article Section 3.1.: Figure 4d). The mirror sections with wide-spaced setae sockets were found in the center of the particular mirrors, but in L4, these were occasionally close to the edges of the mirrors. Approximately 20 % of the mirror area in the respective L4 samples had wide-spaced setae sockets. In L6, the areas of wide-spaced sockets occurred predominantly on the hind-mirrors. The sockets had slightly larger diameters and carried larger setae than those in the sections of close-set sockets (visual check, not measured; see article Section 3.1.: Figure 4e).

Figure S1. SEM-photos of OPM mirrors with squares of 25 µm × 25 µm each for counting of the setae sockets along a 100 µm ∗ 100 µm grid; A—segment 11 of L4; B—segment 11 of L6; the setae were removed mechanically before SEM.

See next page.

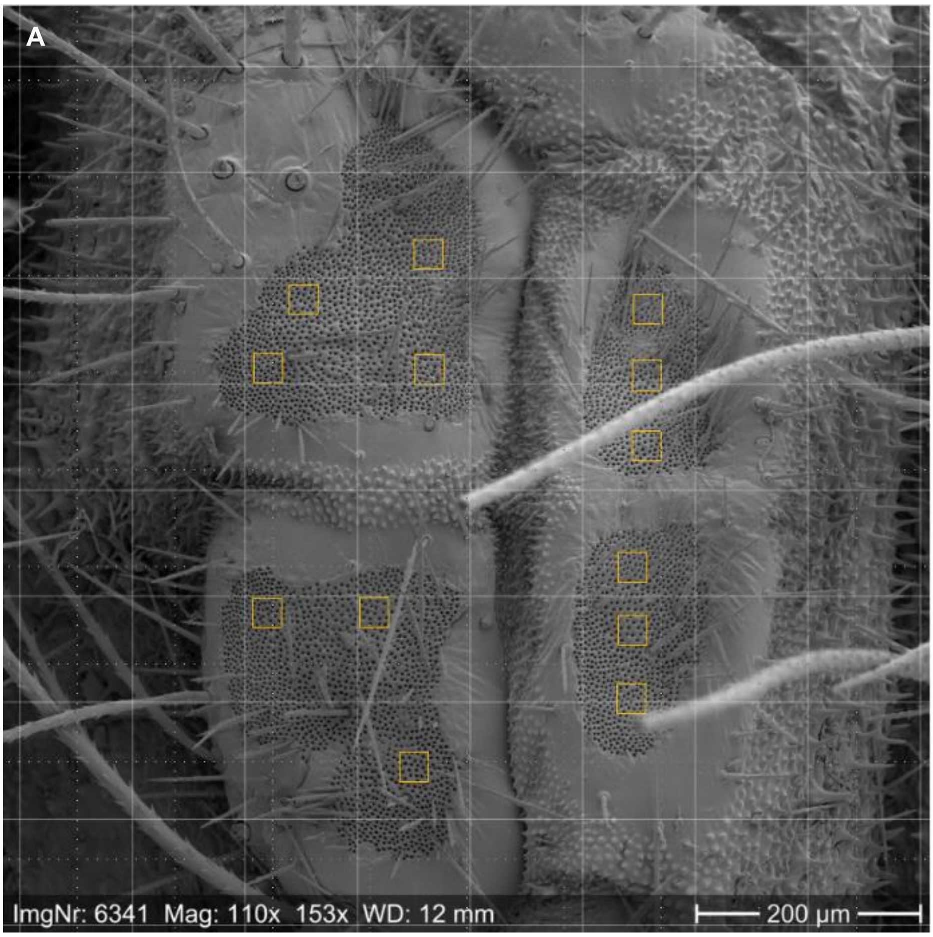
A
ImgNr: 6341 Mag: 110x 153x WD: 12 mm
200 µm

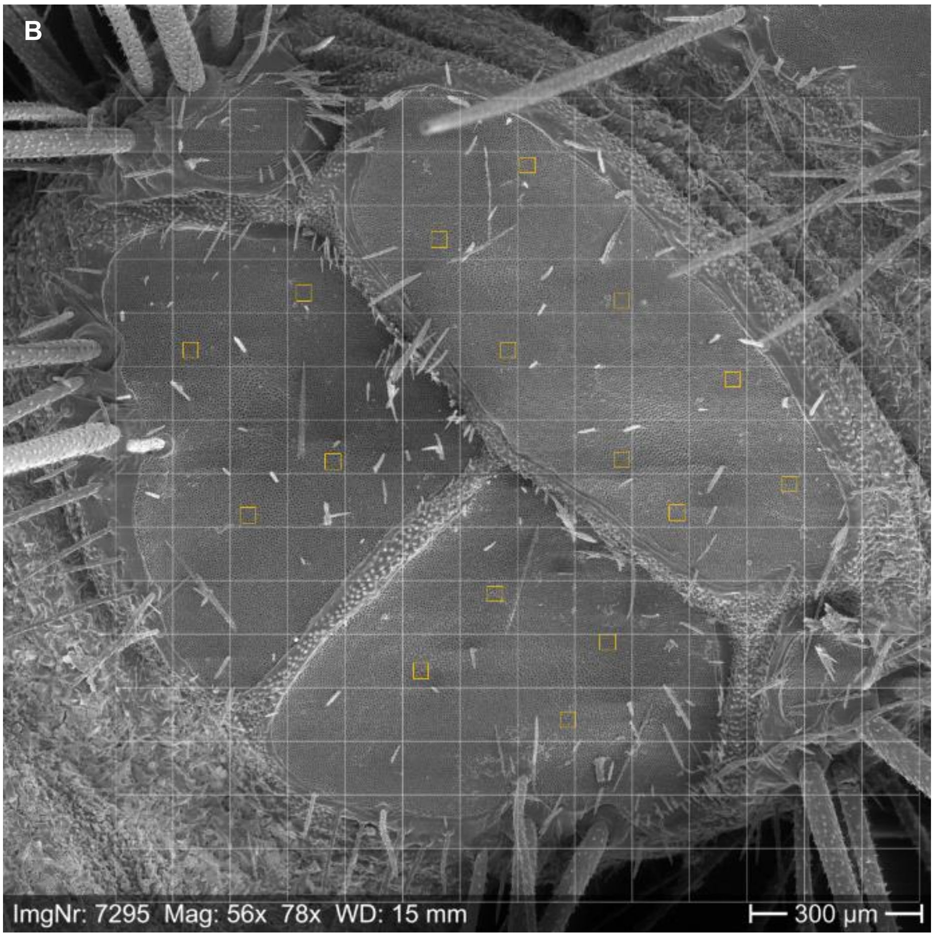
B
ImgNr: 7295 Mag: 56x 78x WD: 15 mm
300 µm

2.3 OPM pupation tent volume

2.3.1 Setae in pupation tents

Before OPM pupation, when the L6 larvae enter the prepupal phase, they shed setae from the mirrors to weave them into the cocoons (Fig. S2). The setae give the cocoons the reddish-brown color.

Besides processionary species, active dislodging of setae was also observed in tarantulas which incorporate the setae into their molting webs and eggsacs [35] and references therein.

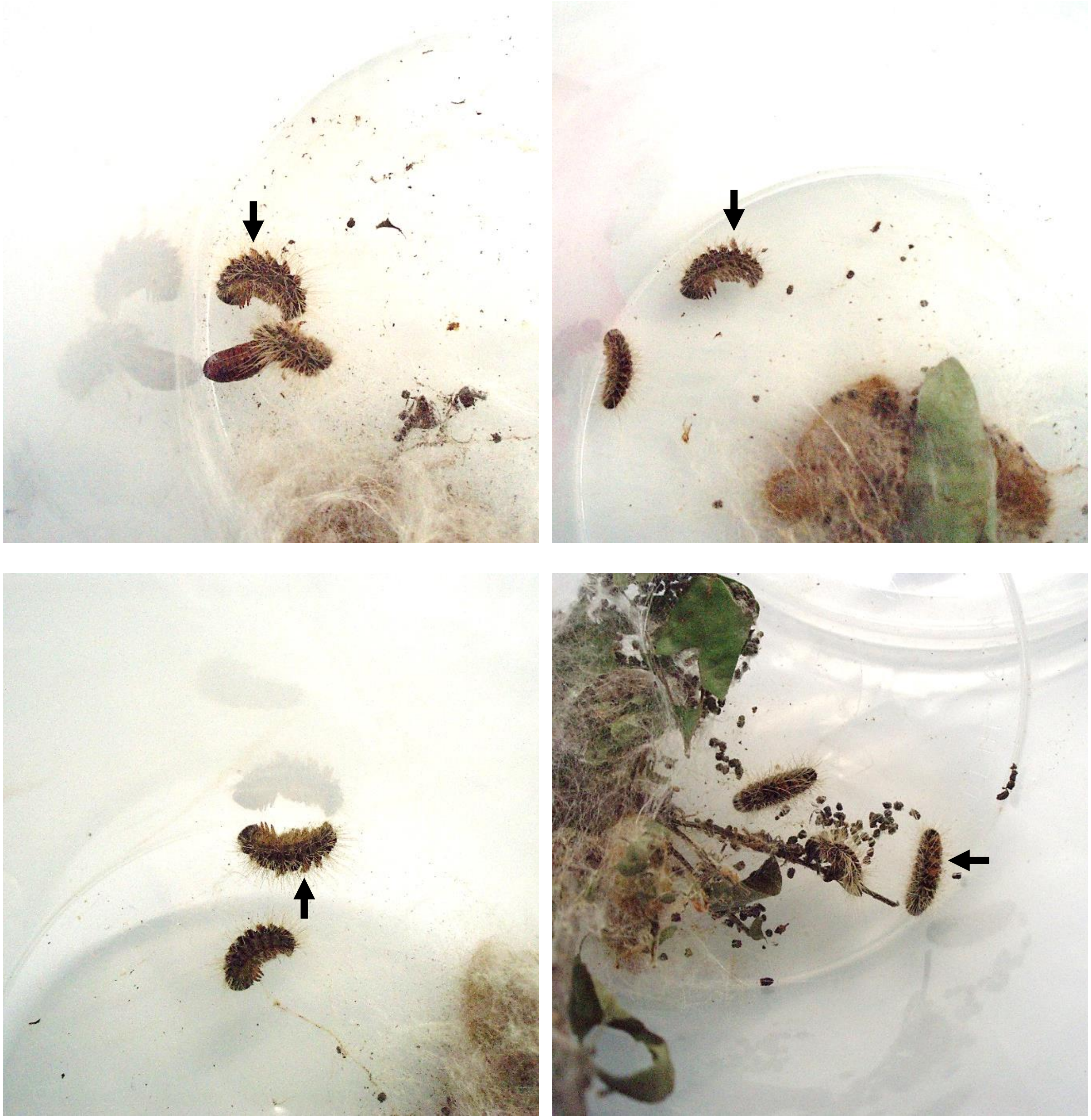

Figure S2. OPM L6 larvae (almost prepupae) in laboratory-rearing on oak leaves shed setae (reddish-brown color; arrows) from the mirrors before pupation.

Details on the rearing method and results, see Halbig et al. [57].

2.3.2 Volume range and number of individuals

The raw data for computing the volume of the OPM pupation tents ("nests") and the number of OPM individuals per tent are shown in Tab. S3 and Fig. S3. The results indicate that the space requirement per individual in the tents is 1.1 ± 0.3 ml (mean ± SD).

Table S3. OPM pupation tent volume and number of individuals per tent.

Tent ID	Diameter (cm)			Volume (liter)	OPM individuals	
	A	B	C		Number	Number per liter tent
1	18	6	8	0.452	565	1249
2	17	9	5	0.401	469	1171
3	5.5	7	3	0.060	35	579
4	4	6	8.5	0.107	70	655
5	2	6	3.5	0.022	19	864
6	13	6	4.5	0.184	203	1105
7	10	5	5	0.131	149	1138
8	9	5	4	0.094	115	1120
**Mean**						**998**
**SD**						**246**

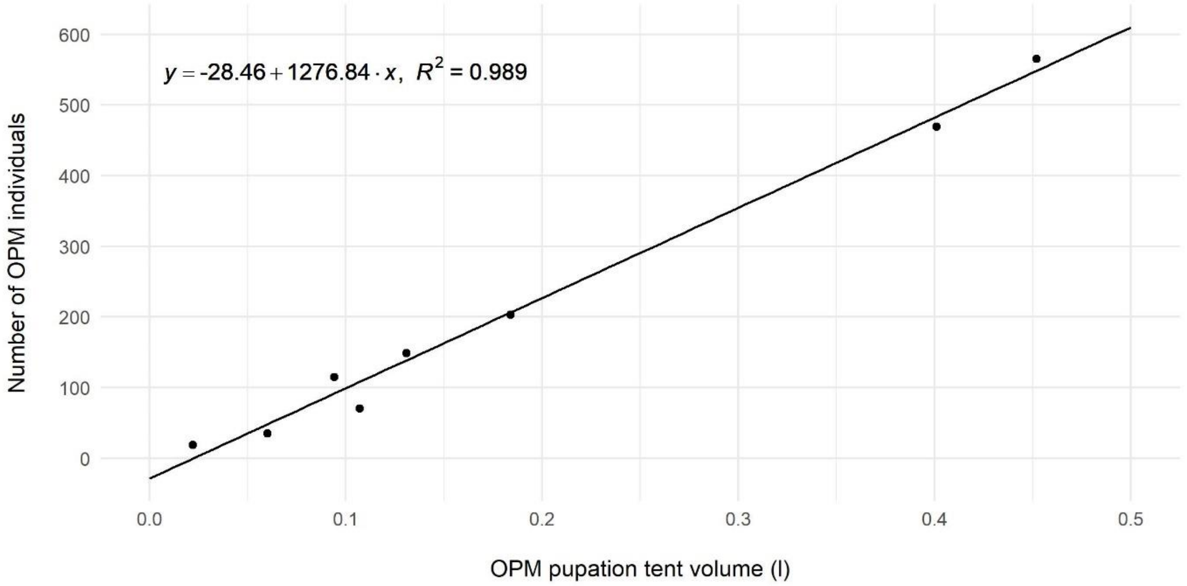

Figure S3. Number of OPM individuals per pupation tent, depending on tent volume (liter).

In addition to our own data giving an exemplary insight (Tab. S3, Fig. S3), data from Mühlfeit et al. [52] were also included in the extrapolation of the setae contamination potential emanating from OPM colonies and infested trees. Mühlfeit et al. [52] examined 62 OPM pupation tents systematically, which were collected at nine different sites in Germany in the years 2016-2019. The mean diameters of the tents (assuming the shape of a tri-axial ellipsoid) were 13.1 cm, 9.3 cm and 4.4 cm; however, these had high variability. The minimum tent

volume was 3 cm^3, while the maximum was 3000 cm^3. The median was 147 cm^3. In total, 24 % of the tents had a maximum size of 50 cm^3, and the majority (48 %) had a volume between 130 cm^3 and 500 cm^3. Only three tents (5 %) were larger.

The number of individuals per tent rises with an increasing tent volume, ranging from 10 to 410 individuals in this study. The highest variability extending from 50 to 380 individuals per tent was found in tent sizes from ca. 100 cm^3 to 1000 cm^3. A tent volume of 1000 cm^3 (1 liter) corresponded to approximately 400 individuals [52].

2.3.3 Pupation tent size classification

The estimation of the total volume of pupation tents per tree is a standard method of OPM population density assessment, for example, in Germany and France (cf. methods used in the United Kingdom: [104]). It is considered the most reliable compared to other methods such as counting of egg batches and pheromone trapping of the adults (own observations, see also [4,105]). The number of egg batches is usually counted in winter on a defined number of twigs of a certain length from the upper crown periphery of a representative number of oak trees. This parameter might be less informative for the following season because of the influence of spring weather on larval development and survival (own observations, [54]). Pheromone trapping targets exclusively male moths, and the results depend on the lure quality and the weather conditions such as precipitation, wind direction and air temperature [105], cf. [106]. The tent volume, however, includes male and female OPM individuals and is directly linked to the releasable setae quantity of larval colonies.

Depending on the oak forest area, a representative number of dominant trees (Kraft's class 2), evenly distributed over the forest stand is selected for counting of OPM pupation tents each year. Keeping the total number of trees, different trees are surveyed in different years. The number of pupation tents per tree (see also [107,108]) and their size is determined by means of binoculars from the ground when the tents are spun at the end of June or the beginning of July. Only fresh tents are considered, which are grey-colored and distinguished from old tents of brownish color from previous years.

The tent size is classified, and the corresponding volume in liters is estimated based on the size of reference objects:

- Small: 0.2 L (golf ball to orange size);
- Medium: 3.0 L (cf. small rugby ball, or size 3 handball: 3.5 L);
- Large (up to 0.5 m length): 6.0 L (cf. size 5 soccer ball 5.5 L);
- Extra-large (more than 0.5 m length): 12 L.

By multiplying the number of tents per tree by their volumes, the tent volume per tree is computed. The volume of molting tents (containing only exuviae) and pupation tents from previous year(s) is not considered.

2.3.4 Whole-tree tent volume

The data of the whole-tree tent volume were obtained from an oak forest near Michaelisbruch in Brandenburg, Germany (for geographic coordinates, see Tab. S1). In this forest, which is part of the nature reserve “Prämer Berge”, protected since 1967, OPM had an outbreak in the years 2016-2019. The oak forest of 4.3 ha is located on two ridges around an opening of 0.5 ha in the center. The tree age was ca. 90 years. Oak (*Q. robur, Q. petraea*, and hybrid oak *Q.* x *rosacea*) was mixed with Scots pine (*Pinus sylvestris*) and small-leaved linden (*Tilia cordata*). During our studies of the airborne OPM setae dispersion in 2016-2019 (unpublished), a total of 30 oak trees (32 in year 2016) from the stand center and the stand edge were examined each year. The tree height was 13 ± 4 m, and the DBH was 40 ± 15 cm (mean ± SD).

Depending on OPM population density, the size of the pupation tents varies. Especially during outbreaks, OPM larvae of several colonies aggregate and form larger tents (own observations, [104]).

The mean tent volume per tree was 28 l (± 20 l SD and ± 4 l SE) at Michaelisbruch in 2017 at the point of culmination of the local OPM outbreak, with a minimum of 3 l and a maximum of 85 l (Fig. S4, Tab. S4). In 2018, the maximum tent volume per tree was 121 l. This outlier resulted from one tree of 18 m height and 80 cm DBH with several large and extra-large tents.

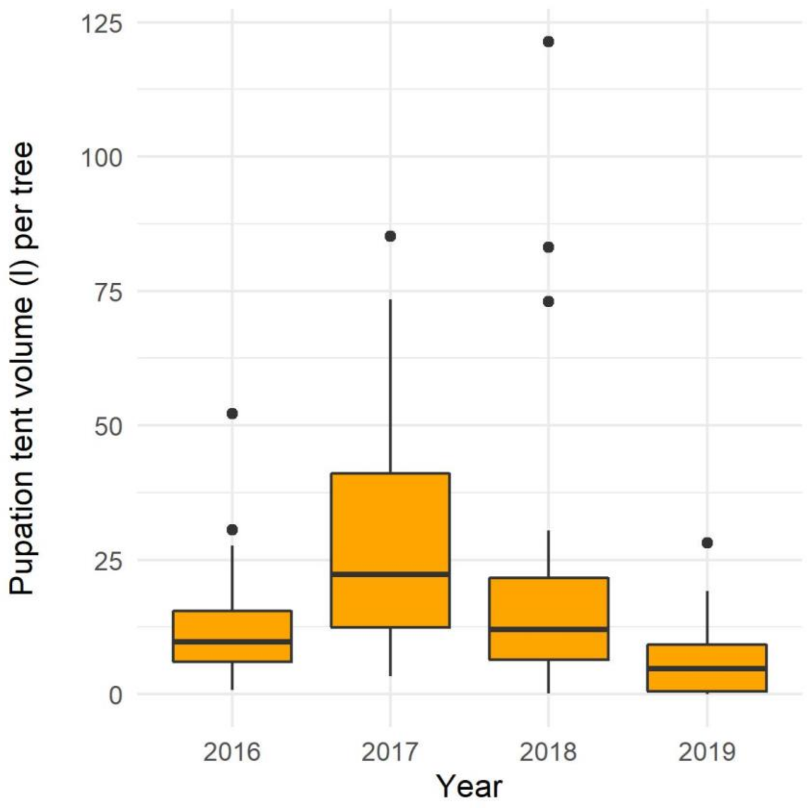

Figure S4. Volume (liter) of fresh OPM pupation tents per tree at Michaelisbruch during an outbreak in 2016-2019.

Table S4. Volume (liter) of fresh OPM pupation tents per tree at Michaelisbruch during an outbreak in 2016-2019; statistical parameters; n—sample size, SD—standard deviation, SE—standard error, CI—95 % confidence interval, lower—lower limit, upper—upper limit, Min—minimum, Max—maximum, Q1—1st quartile, Q3—3rd quartile.

Year	n	Volume (liter) of fresh OPM pupation tents per tree										
		Mean	SD	SE (mean)	CI lower	CI upper	Min	Max	Median	SE (median)	Q1	Q3
2016	32	12.3	10.9	1.9	8.6	16.1	0.8	52.2	9.7	2.4	6.0	15.5
2017	30	28.3	20.3	3.7	21.0	35.5	3.4	85.2	22.3	4.7	12.5	41.1
2018	30	20.5	26.1	4.8	11.2	29.9	0.2	121.4	12.1	6.0	6.4	21.7
2019	30	6.4	6.6	1.2	4.1	8.8	0	28.2	4.8	1.5	0.5	9.2

3 Supplementary data 3—OPM laboratory-rearing on oak-free diets

3.1 Methods

Two experiments of rearing OPM larvae on diets free of oak leaves (of any dosage form) or other substances obtained from oak trees were conducted in the laboratory of FVA.

A food choice experiment with OPM neonates from Fénétrange, France, was performed (for geographic coordinates, see Tab. S1). The egg batches were collected on 28 January 2015, sent cooled to FVA and kept outdoors until 4 February 2015, when they were transferred to Petri dishes at room temperature. The L1 larvae hatched after 11-14 days. Their feeding preference was compared between a wheat germ-based diet, according to Keena et al. [109], and a bean-based diet, according to Bergomaz & Boppré [110], which were provided simultaneously in each Petri dish. In addition to the diet, small pieces of moistened cellulose tissue were put into the Petri dishes to supply the larvae with water.

Another experiment was conducted with 15 egg batches collected near Grezhausen, Germany (for geographic coordinates, see Tab. S1). Only one third of each egg batch was used because the other parts were examined in other studies (unpublished). After a four-week treatment of 10 °C applied in the laboratory from 18 January 2016 onwards, the neonates hatched on 22 February. Subsequently, they were reared on the diet described below (Tab. S5) in Petri dishes in an incubator (ICH 260L, MEMMERT GmbH & Co. KG, Schwabach, Germany) at constant ca. 20.5 °C, ca. 51 % relative humidity and 8:16 h L:D photoperiod from 22 February onwards (Fig. S4).

The diet was prepared modified after Keena et al. [109] by BOKU IFFF staff for rearing of *L. dispar* (Tab. S5). On the same charge of this modified diet, *L. dispar* was reared successfully at BOKU IFFF in 2016.

Table S5. Formulation of the diet with the best rearing result for OPM larvae.

Formulation modified after Keena et al. [109]; BIO-SERV, Flemington, NJ, USA.

Amount (g)	Ingredient
814	Water, distilled (ml)
15	Agar, finely granulated
120	Wheat germ
25	Casein
5	Ascorbic acid (vitamin C)
8	Bio-Serv® Wesson Salt without iron (cool storage)
2	Sorbic acid
1	Methyl paraben
0.1	Chlortetracycline-HCl
10	Bio-Serv® vitamin mixture (cool storage)
0.1	Ferric citrate

During rearing, the larvae were macroscopically examined for the formation of setae in the respective mirrors. After rearing, the setae distribution in the mirrors of the larvae and exuviae was additionally checked with a binocular reflected light microscope (NIKON SMZ 1500, connected to camera NIKON DS Fi1; NIKON CORPORATION, Tokyo, Japan).

3.2 Results

In the food choice experiment, all OPM larvae preferred the bean-based diet and some molted to L2. However, further development was impossible, and the larvae died.

In the experiment in which the prepared diet, modified after Keena et al. [109], was used, all OPM colonies survived and developed to the L3, L4 and L5 instars (Fig. S5, Tab. S6). Rearing was terminated after four weeks on 24 March 2016, before the larvae reached the L6 instar, irrespective of the given larval vitality sufficient for further development.

Macroscopic and microscopic examination of the larvae reared on the diet showed the formation of setae in the respective mirrors (Fig. S5C-E). By comparison, in laboratory-rearing on oak leaves at 20 °C, the development time was two weeks from L1 hatching to L3 and four weeks from L1 hatching to L5 [57].

However, regarding all instars from L1 to L5, the larvae from the diet were generally smaller than the larvae which fed on oak leaves (*Q. robur*), examined in other studies by Halbig et al. [57]: in the laboratory (oak twigs in water vials) and in the field; and in semi-field (rearing on potted oaks; unpublished). These differences in larval body size and head capsule width were visible to the unaided eye, but the length of the larval body and the head capsule width were not measured exactly in these experiments.

The proportion of larvae of the different instars per sample (Petri dish) was estimated, except for the sample IDs 4, 7 and 12, of which the larvae were counted exactly (Tab. S6).

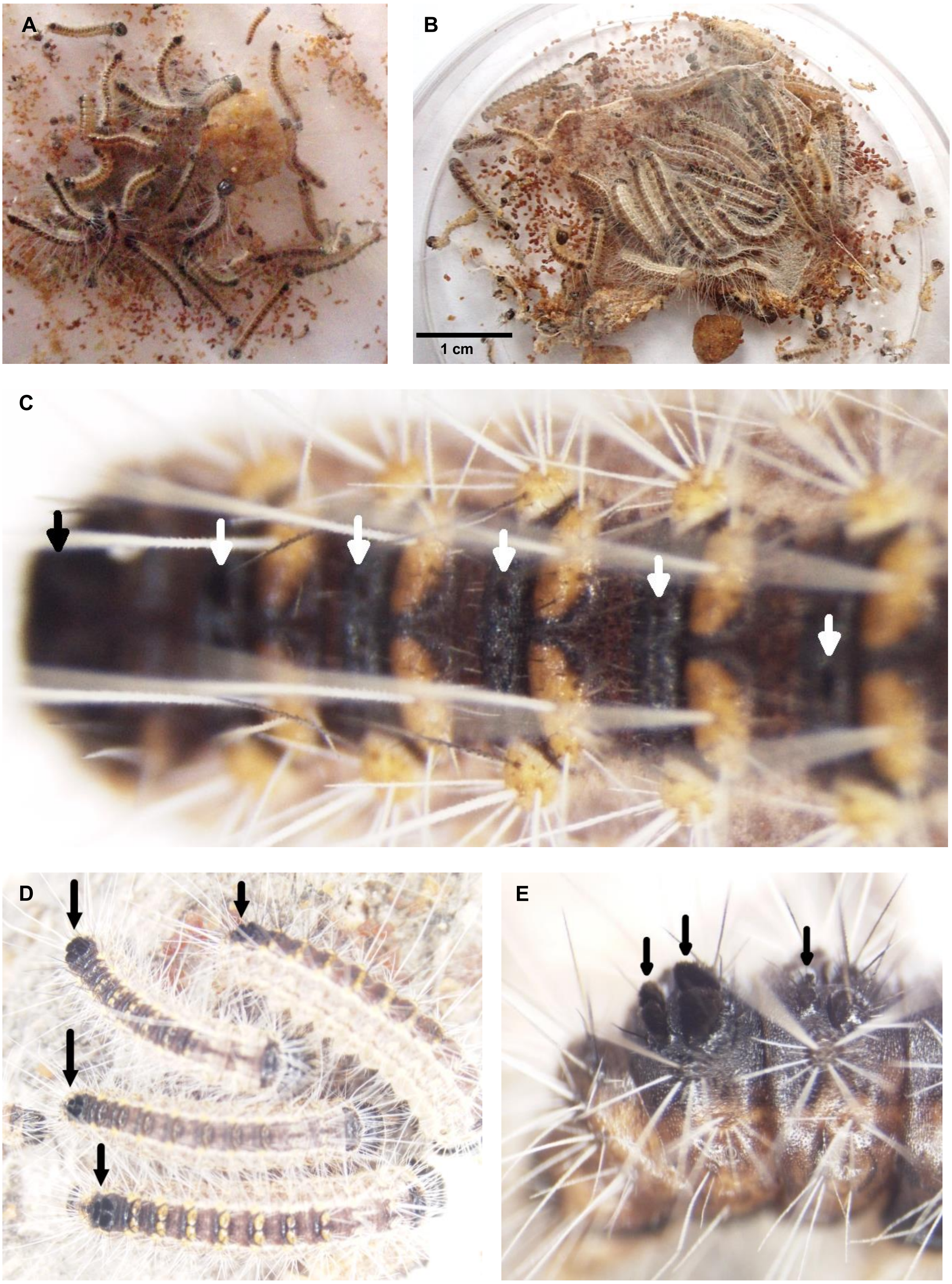

Figure S5. OPM rearing on an oak-free diet in the laboratory in 2016; **A**—feeding larvae; **B**—larvae molting successfully; setae formation by larvae reared on the diet: **C**—setae on the hind-mirrors of the segments 6-10 and on the fore- and hind-mirrors of segment 11 of an L5 larva; **D**—setae (arrows) on the fore- and hind-mirrors of segment 11 of L3 larvae; **E**—setae (arrows) on the hind-mirrors of segment 10 and on the fore- and hind-mirrors of segment 11 of an L4 larva.

Table S6. OPM samples from laboratory-rearing on the oak-free diet.

Diet ingredients (see Tab. S5); all samples contained larvae and exuviae of the preceding instars; NA—no additional information.

<table>
<tr><th rowspan="2">Sample ID</th><th colspan="3">OPM instar (number or proportion of specimens per sample)</th><th rowspan="2">Information</th></tr>
<tr><th>L5</th><th>L4</th><th>L3</th></tr>
<tr><td>1</td><td>> 50 %</td><td>< 50 %</td><td>0</td><td rowspan="4">NA</td></tr>
<tr><td>2</td><td>75 %</td><td>25 %</td><td>0</td></tr>
<tr><td>3</td><td>> 50 %</td><td>< 50 %</td><td>0</td></tr>
<tr><td>4</td><td>0</td><td>0</td><td>5</td></tr>
<tr><td>5</td><td>1</td><td>100 % (except 1)</td><td>0</td><td>L4 larvae molting to L5</td></tr>
<tr><td>6</td><td>0</td><td>2</td><td>100 % (except 2)</td><td rowspan="6">NA</td></tr>
<tr><td>7</td><td>5</td><td>4</td><td>1</td></tr>
<tr><td>8</td><td>50 %</td><td>50 %</td><td>0</td></tr>
<tr><td>9</td><td>0 %</td><td>75 %</td><td>25 %</td></tr>
<tr><td>10</td><td>0</td><td>100 % (except 2)</td><td>2</td></tr>
<tr><td>11</td><td>0</td><td>100 %</td><td>0</td></tr>
<tr><td>12</td><td>2</td><td>8</td><td>5</td><td>L3 died</td></tr>
<tr><td>13</td><td>0</td><td>100 %</td><td>0</td><td rowspan="3">NA</td></tr>
<tr><td>14</td><td>0</td><td>100 %</td><td>0</td></tr>
<tr><td>15</td><td>0</td><td>100 %</td><td>0</td></tr>
</table>

3.3 Discussion

To be independent of seasonal food supply during OPM rearing and to test the hypothesis that the source of food might influence the setae formation, a new laboratory-rearing method for OPM was established. For this purpose, a diet containing no oak leaves or other oak-derived substances was used (for ingredients, see Tab. S5). This method is less effortful than the laboratory-rearing procedure on fresh oak leaves, which was first developed by Halbig et al. [57]. The oak-free diet has been proven to be suitable for rearing OPM larvae which produced setae in this study (Fig. S5). However, the larvae were smaller than those from laboratory-rearing on fresh oak leaves or from the field. Rearing on diets should be tested further with OPM larvae from other geographic origins, i.e., beyond Southwest Germany and Northeastern France, and with adapted diet formulations to verify the results.

4 Supplementary data 4—Airborne setae dispersion

4.1 Technical equipment and study setup

4.1.1 Active samplers

Volumetric samplers similar to the volumetric Hirst–Burkard spore traps [87,88] were used, which were produced by AMET (Velké Bílovice, Czech Republic; Fig. S6C-D).

A volumetric sampler is a cylindrical motorized gadget which absorbs ca. 10 l of air per minute, corresponding to human air inhaling at rest [87]. The air is absorbed through an orifice of defined size at the front of the sampler. The sampler is pivoted and equipped with a wind vane on the back. In the inside of the sampler, a clockwork twirls a plastic disc before the orifice. One rotation takes 7 days (168 h). The plastic disc with 377 mm circumference was equipped with 19 mm wide sticky tape (Scotch® Magic™ tape, 3M COMPANY, Maplewood, MN, U.S.) coated with petroleum jelly along its entire circumference (Fig. S6D). All absorbed airborne particles adhere to the tape. Their settling time can be determined up to an accuracy of approximately ± 1 h, which enables the analysis of the daily cycle and the correlation with the data of the abiotic factors.

In this study, the energy supply for sampler operation in the field was ensured by a photovoltaic panel connected to a rechargeable battery.

4.1.2 Pivoted passive samplers

The pivoted passive samplers used in 2015 were self-made constructions and based on the successful application of similar samplers in Bavaria (Lobinger personal communication 2015; see also [86]). The vertical microscope slides were put into pivoted plastic beakers (customary, conical, 0.2 l) with a cut-out bottom, equipped with a wind vane (Fig. S6B). For adhesion of airborne setae, the upwind side of the slides was coated with petroleum jelly.

4.1.3 Sampler arrangement

Fig. S7 shows an aerial photo of the experimental setup at the study site Klingenbach, Austria (for geographic coordinates, see Tab. S1). Fig. S8 displays photos of the experimental setup of the exposure simulations at Klingenbach in 2014 and 2015, as well as captured setae under the microscope.

4.1.4 Weather station

At the study site, a weather station was installed on a trestle at 2 m agl (BOGNER & LEHNER MESSTECHNIK GMBH, Sankt Marien, Austria; (Fig. S6A). It consisted of sensors for air temperature and relative humidity, which were sheltered by a Stevenson screen, and a cup anemometer to measure wind direction, wind speed and maximum gust speed. Hourly, the wind direction frequency in min/h and the wind speed in m/s were logged for the eight wind direction classes N, NE, E, SE, S, SW, S, and NW.

Data from the weather station on site were used in the analyses of the experiments in August 2014 and June 2015. Additionally, and for the analysis of the preliminary study in June 2014, data from the nearby weather station Mattersburg, Austria, at 10 km distance to the study site Klingenbach, were obtained (for geographic coordinates, see Tab. S1 and Tab. S2). The data from both weather stations were comparable, as a check for August 2014 revealed (data not shown).

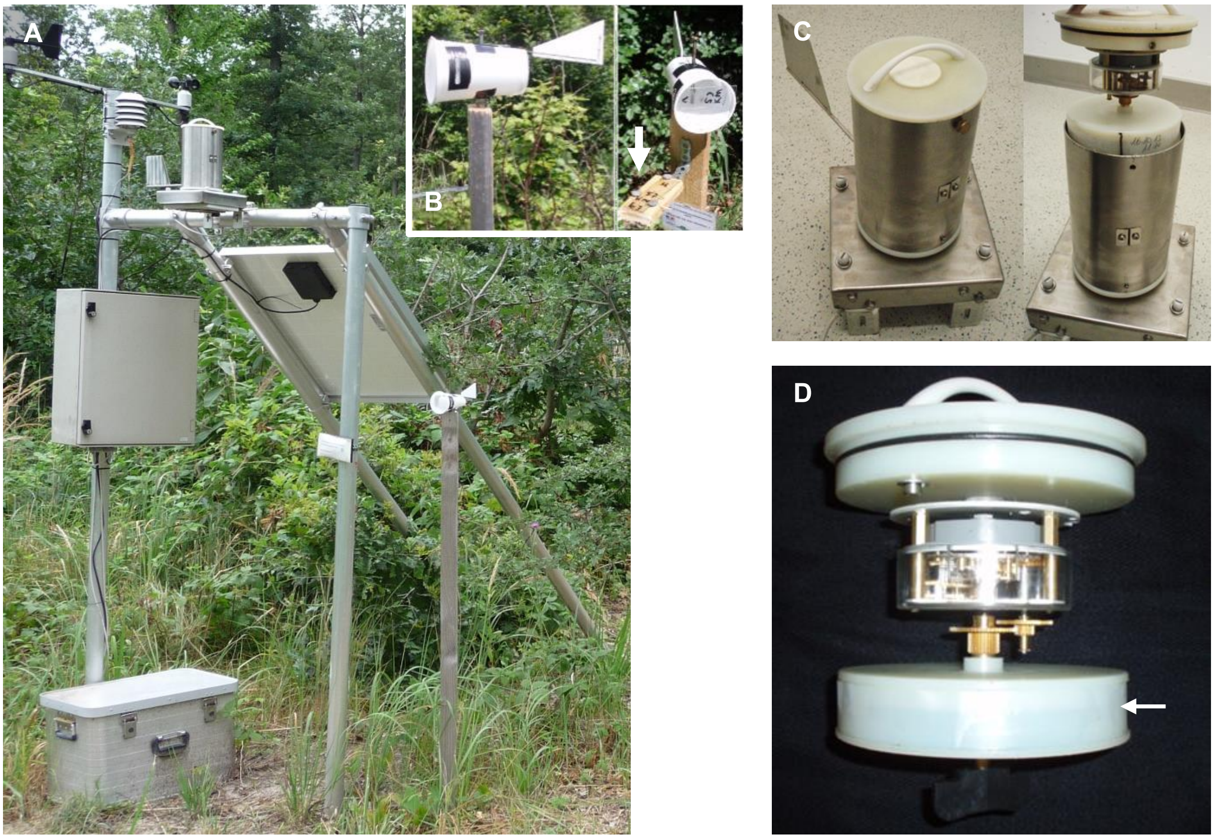

Figure S6. Active and passive samplers used in the airborne setae exposure simulation.

A—weather station, active sampler and photovoltaic panel on a trestle at ca. 2 m agl and pivoted passive sampler at 1.5 m AGL (LTR); **B**—pivoted vertical passive sampler and fixed horizontal passive sampler (arrow); **C**—active sampler closed (left) and opened (right); **D**—inner cylinder of an active sampler with clockwork and rotative disk with adhesive tape (arrow).

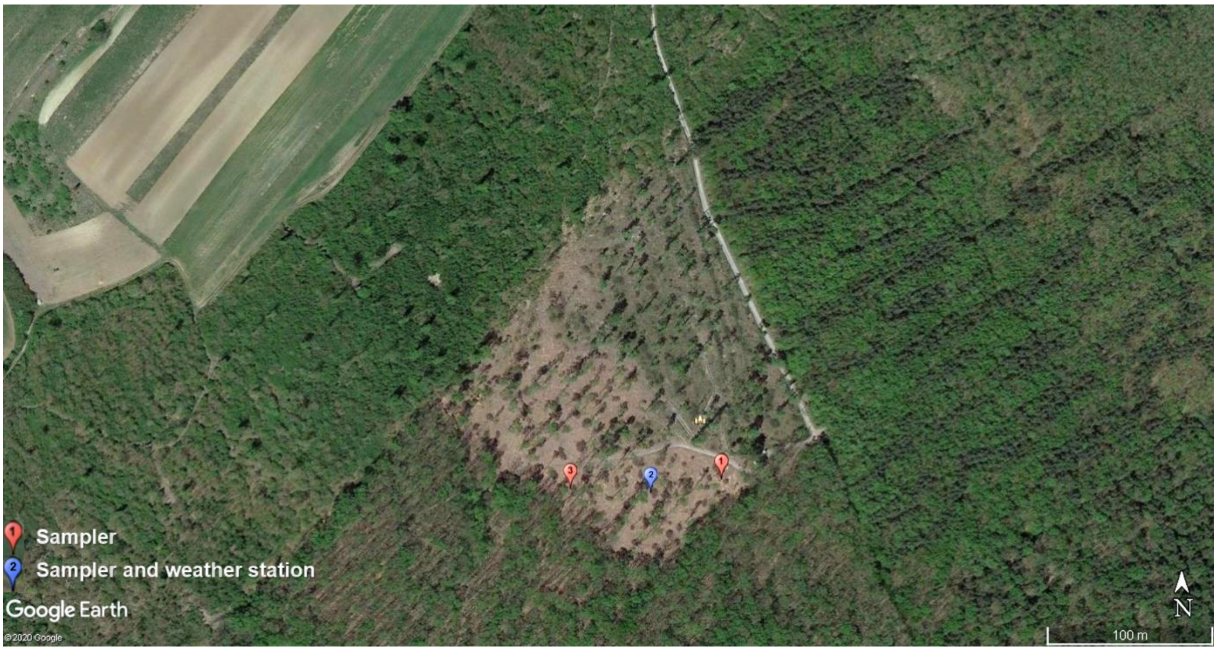

Figure S7. Positions of the samplers and the weather station at Klingenbach during the exposure simulations.

Preliminary study—pairs of active and passive samplers ID 1-3, for details, see Supplementary Data 4.3; study of close range, circular setae dispersion—only active and passive sampler ID 2 were used (passive samplers in circular formation and dummy not shown); study of horizontal setae dispersion—only active and passive sampler ID 1 were used (passive samplers in rows not shown).

Figure S8. Experimental setup of the airborne setae exposure simulations at Klingenbach.

A—Trial 1 of the preliminary study: active sampler ID 2 (arrow), active sampler ID 3, setae source of OPM tents in white mesh bag, blower (LTR);

B—Study of close range, circular dispersion: blower, setae source of OPM tents in blue mesh bags, circles of 11 passive samplers each, dummy, active and passive sampler (FTB), photo: Josef Pennerstorfer;

C—Study of horizontal setae distribution: passive samplers, active sampler with photovoltaic panel, setae source of OPM larvae and tents in green mesh bags, blower (LTR);

D—Setae of different lengths on a slide of the passive samplers at binocular reflected light microscope (NIKON SMZ 1500 connected to camera NIKON DS Fi1, NIKON CORPORATION, Tokyo, Japan), background: 1 mm × 1 mm grid of graph paper.

See next page.

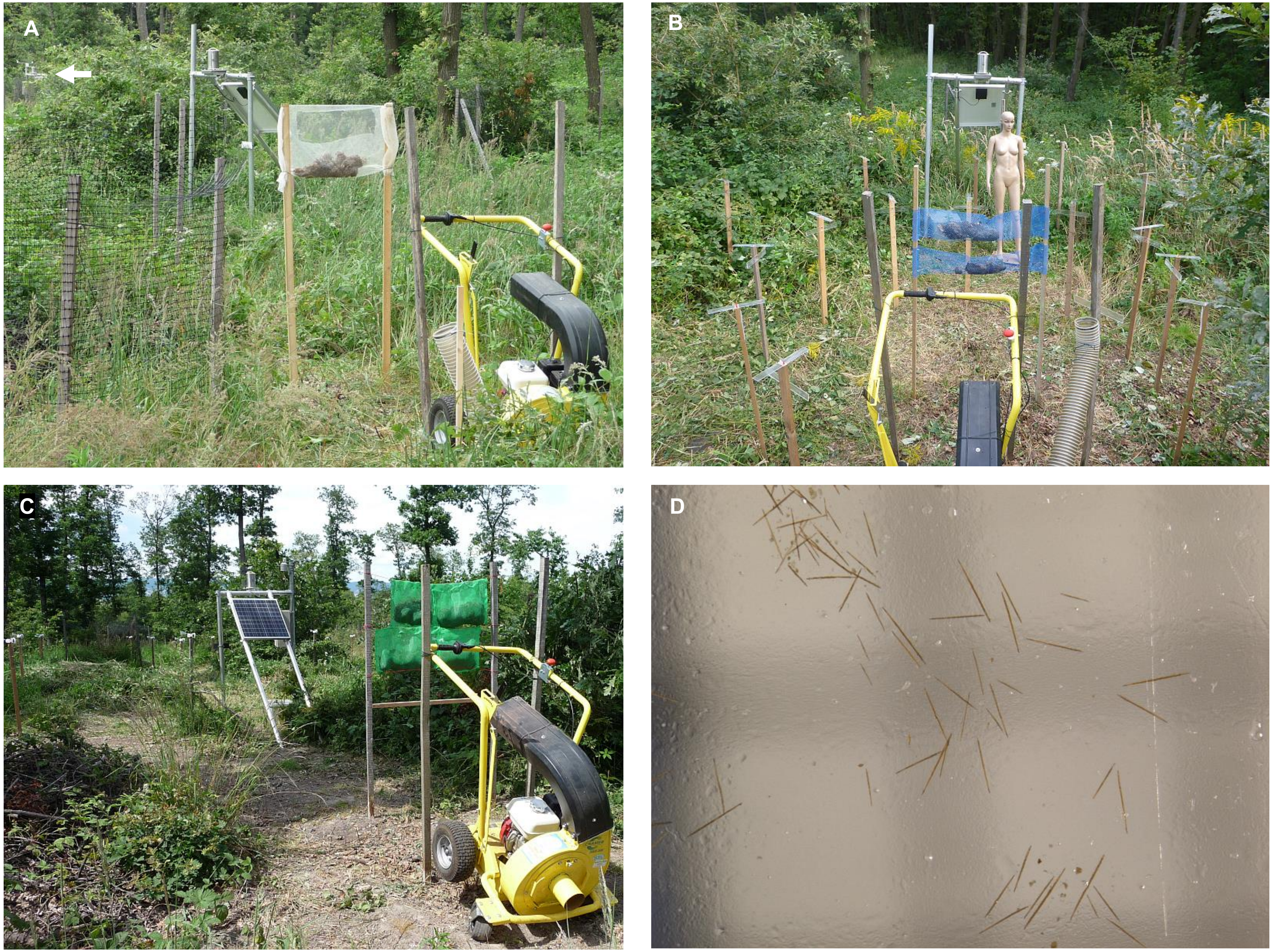
A
B
C
D

4.2 Analysis

4.2.1 Sampler screening

The entire petroleum-jelly-coated surface of the slides and plates of the passive samplers and the respective parts of the active sampler tapes which were exposed during the experiments were screened for setae catch with a binocular reflected light microscope (NIKON SMZ 1500 connected to camera NIKON DS Fi1; NIKON CORPORATION, Tokyo, Japan; Fig. S8D).

4.2.2 Setae source magnitude

The natural OPM population density on site was determined by counting the pupation tents of OPM in the forest adjacent to the opening and at the oak trees bordering the opening in 2014 and 2015, respectively.

After the experiments, the OPM molting and pupation tents or larvae, used as setae source, were frozen at -20 °C for later assessment of the setae source magnitude. This was conducted by counting the OPM individuals and exuviae. After the experiment in 2015, the mesh bags with OPM larvae and molting tents were used in further studies (unpublished). Therefore, counting of the larvae was not possible, but the number of the exuviae in the mesh bags was estimated.

Furthermore, the exuviae were checked for remaining setae, which were not released into the air during the experiments. For this, the mirror area of randomly selected exuviae was examined with a binocular reflected light microscope (NIKON SMZ 1500; NIKON CORPORATION, Tokyo, Japan). Then, the percentage of the area which still carried setae was estimated. Regarding the L5 exuviae, for example, the setae amount on one hind-mirror was estimated corresponding to ca. 5 % of the total setae amount of the exuvia (cf. article section 3.1.: Figure 4c, e, f). The larvae were not considered because they were not translucent, and the mirror area was not sufficiently visible with the microscope for the assessment.

4.2.3 Statistical analysis

Horizontal setae dispersion

Table S7. Statistical parameters of the setae catch in the rows of the large vertical passive samplers in the setae exposure simulation in June 2015; CLD—compact letter display according to the statistical differences (see Tab. S8).

CLD	Distance (m)	Number of setae per cm² sampler					
		Minimum	1st Quartile	Median	Mean	3rd Quartile	Maximum
ab	5	0.03	0.03	0.06	0.11	0.24	0.24
ab	10	0.01	0.02	0.05	0.05	0.08	0.08
bc	15	0.01	0.01	0.03	0.03	0.03	0.09
c	20	0.00	0.00	0.01	0.00	0.01	0.01

Table S8. Probability values of the statistical tests to determine the differences in the setae catch between the rows of the large vertical passive samplers in the setae exposure simulation in June 2015.

P-values adjusted by Benjamini-Hochberg-method; 95 % confidence interval.

Statistical tests	Row distance to the setae source (m)	P-value
Kruskal–Wallis rank sum test	All distances	0.011
Dunn Kruskal–Wallis multiple comparison	5 vs. 10	0.526
	5 vs. 15	0.183
	5 vs. 20	0.018
	10 vs. 15	0.363
	10 vs. 20	0.022
	15 vs. 20	0.122

4.3 Preliminary study

4.3.1 Methods

On 24 June 2014, two setae exposure simulation trials, each lasting for 1 h were carried out. Three pairs of samplers (ID 1-3, each: active and passive) were installed at Klingenbach, Austria (for geographic coordinates, see Tab. S1). Each passive sampler consisted of a horizontal and a vertical microscope slide of 76 mm × 26 mm each, both fixed and not pivoted, which were mounted on roof battens at ca. 1.3 m AGL. The pairs of samplers were arranged in a line transect at 50 m distance to each other (Fig. S7, Fig. S8A, Fig. S9). The air current was created by the blower placed in front of sampler position ID 3.

Two different types of setae source were used in the two trials at two different distances from the blower (Tab. S9). For trial 1, OPM pupation tents were collected in Vienna (Lainzer Tiergarten) and in a forest close to Klingenbach on 24 June 2014. They originated from OPM infestations in previous years. For trial 2, three fresh OPM tents from a private garden in Vienna (Pötzleinsdorf, for geographic coordinates, see Tab. S1), containing living L6 larvae, were used. The tents were put into a mesh bag with a mesh size of 1 mm × 1 mm and installed at 1.5 m AGL in front of the blower. The wind speed was measured with a portable vane anemometer (EXAKT-ASDi, HÖNTZSCH GMBH & CO. KG, Waiblingen, Germany) at different positions before the experiment started:

- Blower orifice 12.6 m/s;
- Before mesh bag 4.0 m/s;
- Behind mesh bag 2.6 m/s;
- Before active sampler ID 3 0-0.6 m/s.

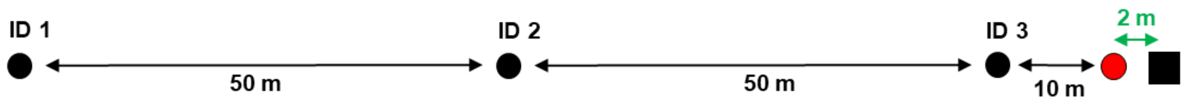

Figure S9. Experimental setup of trial 1 in the preliminary study of the airborne setae exposure simulation.

Black dots—pairs of active and passive samplers, red dot—setae source, black square—blower; diagram is not true to scale.

4.3.2 Results

The setae catch was zero in both trials, apart from two catches (Tab. S9). Therefore, statistical analyses were omitted. The active samplers at the positions ID 1 and ID 2 did not catch any setae. The setae catch by active sampler ID 3 was zero in trial 1 and seven consisting of one single seta and one cluster in trial 2. The only setae catch by the passive samplers was found at the horizontal position ID 2 in trial 1.

The data of air temperature, relative humidity, wind speed and wind direction (Tab. S9) were obtained from the weather station Mattersburg, Austria, at ca. 10 km distance from the study site (for geographic coordinates, see Tab. S1 and Tab. S2).

Air temperature and relative humidity did not vary substantially during the day. They averaged 18.3 ± 1.5 °C and 57.9 ± 6.2 % (mean ± SD) within the period 05:00-20:00 CEST. The daily maximum air temperature was reached during the experiment. There was no rainfall on 24 June 2014.

In both trials, the setae source consisted of L5 larvae and exuviae (Tab. S9). A total of 95 and 23 samples were examined for the remaining setae in trials 1 and 2, respectively. The experimental blowing of the setae source did not release the complete (maximum) setae amount (Tab. S9).

Table S9. Key figures of the experimental setup and the results of the preliminary study of airborne setae exposure simulation.

Key figure		Trial 1	Trial 2
Time (CEST), 24 June 2014		16:15 – 17:15	18:00 – 19:00
Weather	Air temperature	20.4 °C	19.4 °C
	Relative humidity	58.5 %	60.0 %
	Wind speed and direction	0.65 m/s (N, NE)	1.30 m/s (SSE)
Setae source	Distance from the blower	2 m	1 m
	Distance to sampler ID 3	10 m	5 m
	Number of OPM tents	5	3
	Age of OPM tents	≥ 1 year (from previous years)	0 years (fresh)
	Number of OPM larvae and exuviae	50 larvae, 1550 exuviae	100 larvae, 310 exuviae
	Remaining setae (mean ± SD)	22 ± 23 %	6 ± 11 %
Setae catch	Active samplers	0	7 (ID 3)
	Passive samplers	1 (ID 2)	0

4.3.3 Discussion

The low setae catch (one and seven setae) in both trials of the exposure simulation might have been caused by the almost stable atmospheric conditions and low wind speeds. Thus, the air flow from the blower streamed against an almost stagnant air mass or might have even been slightly deflected by natural gentle wind.

Reducing the distance between blower and setae source from 10 m to 5 m raised the setae catch faintly in trial 2. Moreover, fresh OPM tents were used in trial 2, potentially providing more releasable setae than the tents from previous years, used in trial 1.

The prevailing situation of natural wind, plus the artificially created air flow, did not correspond to the conditions on windy days on which the most severe airborne setae contaminations of people occur [21]. The spread distance and the registered concentrations of airborne setae were much lower than expected. Probably, the setae swirled around the source after being hit by the airstream from the blower rather than dispersed straight downwind. But the experimental setup did not allow for the measurement of setae turbulences. The examination of potential turbulent setae spread in horizontal direction was thus focused in the following experiments in August 2014 and June 2015.

Summarizing, the preliminary study gave first insights into airborne setae contamination under simulated exposure and wind conditions. The subsequent studies were based on these findings, particularly regarding the experimental setup, i.e., setae source, spatial distance between blower and setae source, and positioning of the samplers.

4.4 Experimental limitations

Under natural conditions, higher setae contamination and further setae dispersion were observed than in the exposure simulation experiments (own unpublished results). This was probably due to the characteristics of the simulated setae source and wind conditions.

The setae source was limited in size, constitution and exposure. Moving larvae release more setae than immobile tents or exuviae, especially if those originate from previous years or were stored/frozen before use (own unpublished results). Larger amounts of setae might be released from freely moving larvae and from wind-exposed tents by air currents not decelerated by mesh. The wind speed at low height above ground level is lowered by friction on ground and vegetation. Setae from sources higher above ground level are expected to remain in the air for a longer period of time, which ensues in later deposition, spreading over longer distances (cf. [67]).

Furthermore, the created airstream was less powerful than natural wind. At short distances such as 10 m, the airstream attenuated considerably, especially when operating against a stagnant air mass (in the preliminary study). The airstream was temporarily deflected by natural winds (in the following studies). The directional (ray-like) artificial airstream induced turbulences and vertical setae dispersal.
